# Policies for expanding family planning coverage: lessons from five successful countries

**DOI:** 10.3389/fpubh.2024.1339725

**Published:** 2024-05-14

**Authors:** Franciele Hellwig, Laísa Rodrigues Moreira, Mariângela F. Silveira, Carolina Sales Vieira, Paulina Belén Rios-Quituizaca, Marcela Masabanda, Joel Serucaca, Silas Rudasingwa, Alypio Nyandwi, Shegaw Mulu, Hoda Rashad, Aluísio J. D. Barros

**Affiliations:** ^1^International Center for Equity in Health, Federal University of Pelotas, Pelotas, Brazil; ^2^Postgraduate Program in Epidemiology, Federal University of Pelotas, Pelotas, Brazil; ^3^Department of Obstetrics and Gynecology, University of São Paulo, São Paulo, Brazil; ^4^Faculty of Medical Sciences, Central University of Ecuador, Quito, Ecuador; ^5^International Consultant, Quito, Ecuador; ^6^Rwanda Biomedical Centre, Kigali, Rwanda; ^7^African Population and Health Research Center, Nairobi, Kenya; ^8^Ministry of Health, Addis Ababa, Ethiopia; ^9^Social Research Center, The American University in Cairo, Cairo, Egypt

**Keywords:** Brazil, Ecuador, Egypt, Ethiopia, Rwanda, literature review, policy, family planning

## Abstract

**Background:**

Enhancing the design of family planning interventions is crucial for promoting gender equality and improving maternal and child health outcomes. We identified, critically appraised, and synthesized policies and strategies from five selected countries that successfully increased family planning coverage.

**Methods:**

We conducted a policy analysis through a scoping review and document search, focusing on documents published from 1950 to 2023 that examined or assessed policies aimed at enhancing family planning coverage in Brazil, Ecuador, Egypt, Ethiopia, and Rwanda. A search was conducted through PubMed, SCOPUS, and Web of Science. Government documents and conference proceedings were also critically analyzed. National health surveys were analyzed to estimate time trends in demand for family planning satisfied by modern methods (mDFPS) at the national level and by wealth. Changes in the method mix were also assessed. The findings of the studies were presented in a narrative synthesis.

**Findings:**

We selected 231 studies, in which 196 policies were identified. All countries started to endorse family planning in the 1960s, with the number of identified policies ranging between 21 in Ecuador and 52 in Ethiopia. Most of the policies exclusively targeted women and were related to supplying contraceptives and enhancing the quality of the services. Little focus was found on monitoring and evaluation of the policies implemented.

**Conclusion:**

Among the five selected countries, a multitude of actions were happening simultaneously, each with its own vigor and enthusiasm. Our findings highlight that these five countries were successful in increasing family planning coverage by implementing broader multi-sectoral policies and considering the diverse needs of the population, as well as the specific contextual factors at play. Successful policies require a nuanced consideration of how these policies align with each culture’s framework, recognizing that both sociocultural norms and the impact of past public policies shape the current state of family planning.

## 1 Introduction

Universal access to family planning care is now recognized as a critical component of global health and a key driver to the global development agenda (1). Achieving universal coverage of family planning has become a significant priority for many countries, with notable progress made by several of them (2, 3).

While the public debate on family planning started in developed countries at the beginning of the 20^th^ century, mainly centered on eugenics and women’s rights (4), the international focus on family planning in developing countries emerged after World War II when population changes and economic growth began to be recognized as global concerns (4–8). The potential consequences of rapid population growth for international political security and economic development prompted private foundations and governments of high-income countries to finance population policies in low- and middle-income countries (4–6, 9–11). The initiatives received a positive response from some governments, which were also worried about their demographic changes and economic consequences (5). The initial family planning efforts were made through intergovernmental conferences, which laid out plans of action aimed at developing modern contraceptives and reducing fertility rates through the use of sterilization (5, 7, 10, 12–15). After the establishment of the United Nations (UN), the first major international conference was the World Population Conference held in Bucharest in 1974. At the conference, much of the debate was skeptical on family planning programs, with a strong concern that the Western powers were pressing too hard for population control, without giving the necessary importance to a broader approach. Nevertheless, many new initiatives were launched after the Conference, and massive increases in the use of contraceptive methods and decreases in fertility rates were witnessed worldwide (4).

Over time, the evaluation of previous efforts and the evolving socioeconomic, demographic, and cultural landscape shaped the revision and development of public policies to provide access to family planning. A milestone in family planning policies was the 1994 International Conference on Population and Development (ICPD) held in Cairo, where the goal of meeting the needs and improving the quality of life for all was adopted as the path to promote sustainable economic growth (13, 16). The fundamental approaches proposed at the ICPD included the promotion of women’s empowerment, comprehensive reproductive and sexual health services, and increased funding for family planning (13, 14, 17–22).

The importance of family planning was further recognized in the Millennium Development Goals (2000) and the Sustainable Development Goals (2015), with family planning identified as a cost-effective intervention related to good health, well-being, and gender equality (12, 23). Commitments to family planning were renewed with the 2012 London Family Planning Summit and its ensuing FP2030 initiative, and with the 2019 Nairobi Summit, known as ICPD+25 (24–26).

Family planning is a multifaceted strategy encompassing social, cultural, economic, and health components. While numerous family planning policies and strategies have been implemented, some have yielded better results than others. In a previous study we selected six countries from several world regions that presented impressive improvements in increasing family planning coverage and reducing inequalities to identify the conditions that allowed them to achieve progress (27). Countries selected were Afghanistan, Brazil, Ecuador, Egypt, Ethiopia, and Rwanda. The study indicated that family planning coverage increased alongside progress in gender equality, reduction of poverty, and better health intervention coverage among women from more vulnerable groups. Beyond these contextual improvements, several policies and strategies were implemented in these exemplar countries.

In this study, we focus on the political context of the selected countries and identify the policies and programs implemented to identify which were the key strategies adopted that led to success, either specific for a country or common across countries.

## 2 Methods

### 2.1 Selection of the countries

We were interested in policies that intended to increase family planning coverage in countries from diverse contexts, which presented impressive improvements in increasing family planning coverage and reducing inequalities. The five countries included were Egypt, Ethiopia, Rwanda, Brazil, and Ecuador. The Asian country in our previous analysis (27) was Afghanistan, given it was the country with the fastest increase in demand for family planning satisfied among the Asian countries with available data, even with its strong religious and strict social norms (27). However, given its current political situation, we opted to exclude Afghanistan from this analysis.

### 2.2 Review of relevant literature

#### 2.2.1 Literature search strategy

This analysis is composed of two main strategies. The first one is a literature review of peer-reviewed articles and gray literature, in which all types of documents describing or evaluating a policy to increase family planning coverage were eligible. We conducted searches on two occasions: July 8th, 2022, and May 23rd, 2023, to identify peer-reviewed studies. PubMed/Medline, Scopus, and Web of Science databases. The search strategy consisted of a combination of the names of the selected countries, family planning, and intervention keywords. Keywords related to family planning were “*family planning*,” “*contraception*,” and “*reproductive health*.” For intervention keywords, were included “*policy*,” “*strategy*,” “*program*,” “*evaluation*,” “*legislation*,” “*intervention*,” and “*campaign*.” The key terms were combined using Boolean operators with no restriction of language. Details on the search strategy in selected databases can be found in Supplementary material. Gray literature was searched with Google Scholar, Google, World Health Organization,^1^ FP2020,^2^ UNFPA,^3^ United Nations,^4^ and websites of the Ministries of Health of the selected countries. Conference proceedings were also checked, namely the 1994 International Conference on Population and Development and the 2012 London Summit.

Whenever a policy was identified through the documents, a comprehensive approach was undertaken, involving cross-referencing with official documents and evaluations publicly available. To supplement this effort, we also contacted key informants in each of the five countries.

Reports were limited to publications between 1950 and 2023. We opted to use 1950 as the starting date following the foundation of the Population Council and the first efforts to promote family planning in low- and middle-income countries. This timeframe allows us to capture the foundational years, providing a comprehensive view of developments over the following decades.

#### 2.2.2 Selection and data collection process

All documents retrieved from the different databases were stored in Rayyan software and duplicates were removed by a manual revision. For this review, we were interested in interventions that intended to promote family planning coverage. Interventions could be programs, policies, strategies, legislations, regulations, clinical guidelines, financial interventions, campaigns, and networks. All studies that described or mentioned any type of intervention were selected. Workshops, meetings, and conferences were not considered as policies. Documents of such events were screened only to identify tangible interventions resulting from such events. Randomized control trials and other studies proposing new strategies were not included. After the identification of relevant references, a comprehensive review was conducted. The name, type, year of implementation, population target, main objective, and evaluation result of each policy were extracted from these references and organized in a single file. This compiled list was shared with local experts for their input to ascertain the inclusion of all pertinent policies.

#### 2.2.3 Data synthesis

Study findings were presented in a descriptive, narrative synthesis, starting with an overview of the contextual factors of each country and a brief description of their progress in terms of family planning coverage. Subsequently, to identify primary focuses and assess the diversity of interventions across countries, the findings were organized considering essential factors relevant to achieving universal coverage of family planning. These factors encompassed fertility regulation, basic contraceptive provision, community-based distribution, integration of family planning with other services, social marketing, quality of care, policies for adolescents, and considering equity lens. Additionally, we summarized the number and period-range of policies. The more relevant policies were also organized in timetables according to the policy type. Considering the broad period, the timetables were organized into 10-year periods. The list of all the identified policies with their specific year of implementation can be found in Supplementary material.

### 2.3 Data analysis

To illustrate the improvements observed in the selected countries, we also included quantitative analysis of national health surveys, namely Demographic and Health Surveys, Multiple Indicator Cluster Surveys, Reproductive Health Surveys, and non-standard national health surveys.

For each country, we estimated time trends in demand for family planning satisfied by modern methods (mDFPS) at the national level and among the poorest 20% and the wealthiest 20% of the population. The mDFPS is one of the SDG indicators used to track progress in sexual and reproductive health and it is defined as the proportion of women in need of contraception who were using (or whose partner was using) a modern contraceptive. A woman was considered in need of contraception if she was sexually active, fecund, and did not want to become pregnant within the next two years, or if she was unsure about whether or when she wanted to become pregnant. Pregnant women with a mistimed or unintended pregnancy are also considered in need of contraception. Where all the required information to identify women in need for contraception was not available (non-standard surveys from Brazil and Ecuador), mDFPS was estimated from modern contraceptive prevalence using the following predictive equation (28):

$$\mathrm{logit}\left(\mathrm{mDFPS}\right)=0.61+0.68\log\;\left(\mathrm{CPRm}\right)+3.57\;{\mathrm{CPRm}}^{2}$$

Where CPRm is the modern contraceptive use prevalence, defined as the proportion of reproductive-aged currently using a modern contraceptive method. Time trends on CPRm were also estimated and results were included in Supplementary material.

Using data from the oldest and the latest survey, we also estimated the method mix, considering the proportion of modern contraceptive users using a short-acting reversible method (oral contraceptive pills, injections, spermicides, male and female condoms, path, diaphragm, and vaginal ring), a long-acting reversible method (intrauterine devices (IUD) and implants), or a permanent method (male and female sterilization).

## 3 Results

Through the literature search, a total of 5,515 documents were initially identified, out of which 1,960 were found to be duplicates. 231 studies were selected, with 66 delving into actions enacted in Ethiopia, 61 in Brazil, 51 in Egypt, 46 in Rwanda, and 11 in Ecuador. From these searches, 111 policies were identified. 61 additional policies were identified through gray literature research, supplemented by 24 more identified via consultation with field experts.

**Figure 1:**
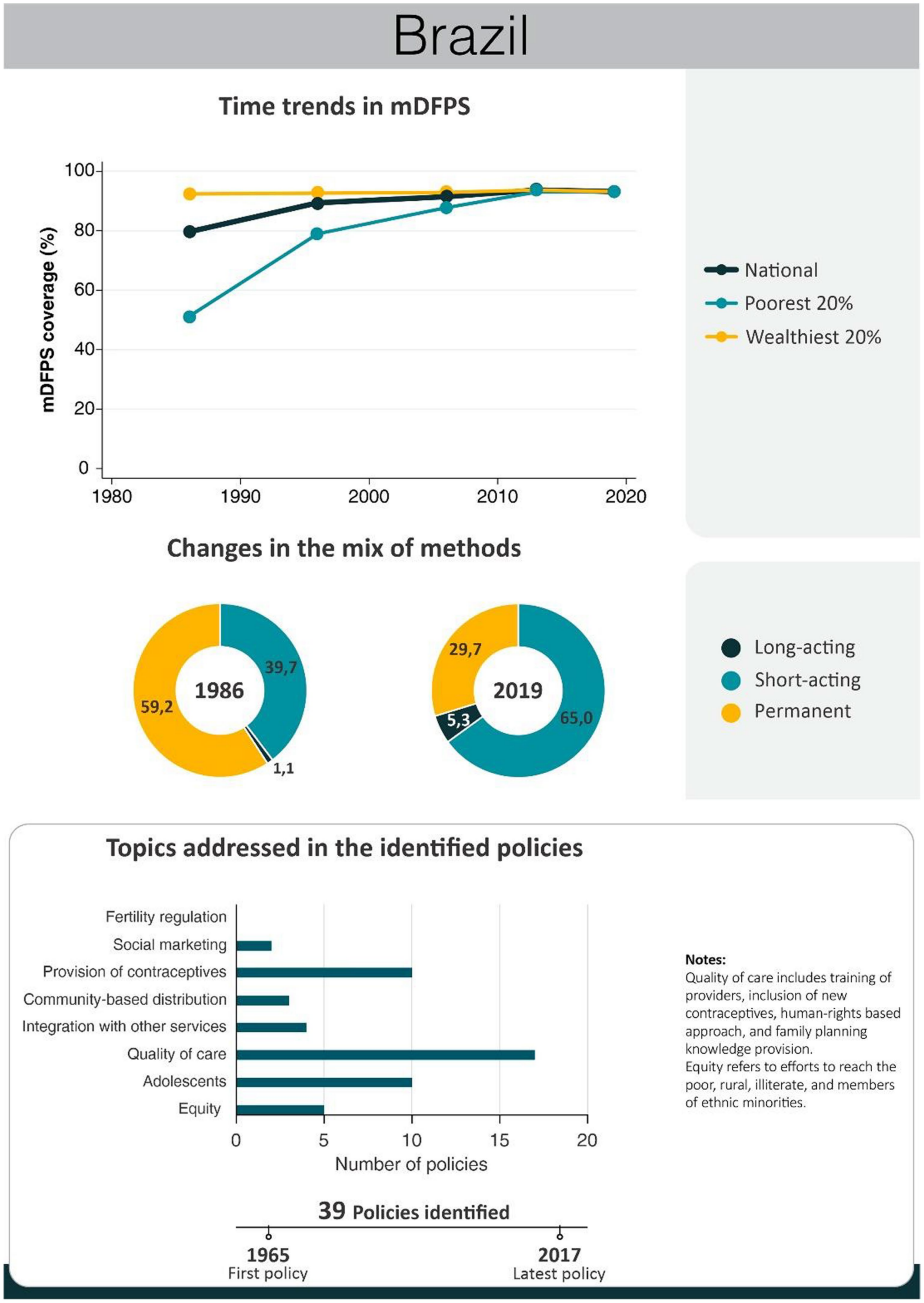
National trends in demand for family planning satisfied by modern methods and among the poorest and the wealthiest women, share of modern contraceptive use in the first and in the last available national health surveys, and topics addressed by policies implemented in Brazil. Contraceptive methods were grouped into reversible short-acting (e.g., pill), long-acting (e.g., IUD) and permanent contraception. Source: Demographic and Health Survey and *Pesquisa Nacional de Saúde*.

**Figure 2:**
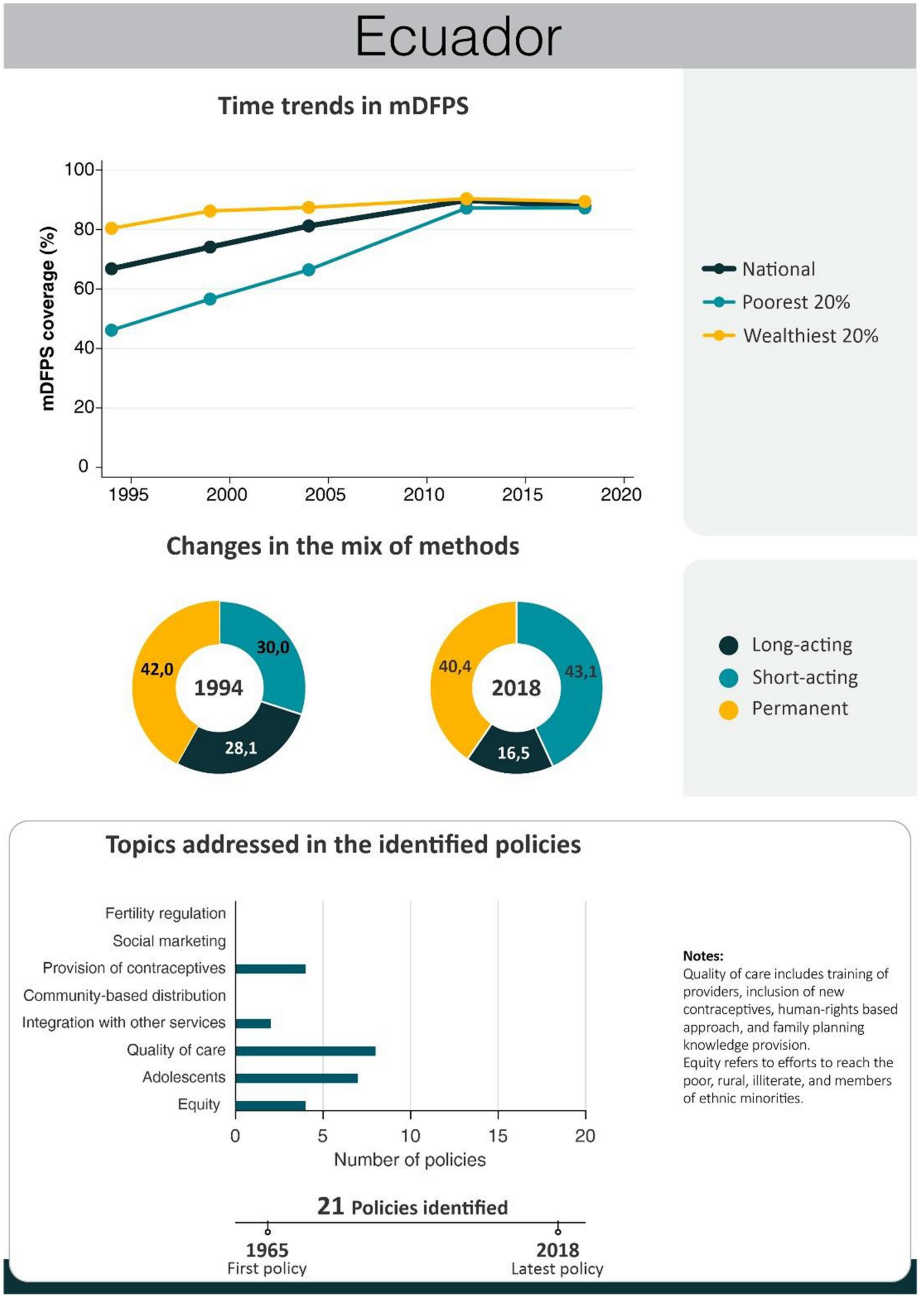
National trends in demand for family planning satisfied by modern methods and among the poorest and the wealthiest women, share of modern contraceptive use in the first and in the last national health survey, and topics addressed by policies implemented in Ecuador. Source: Reproductive and Health Survey, *Encuesta Nacional de Salud y Nutrición*.

**Figure 3:**
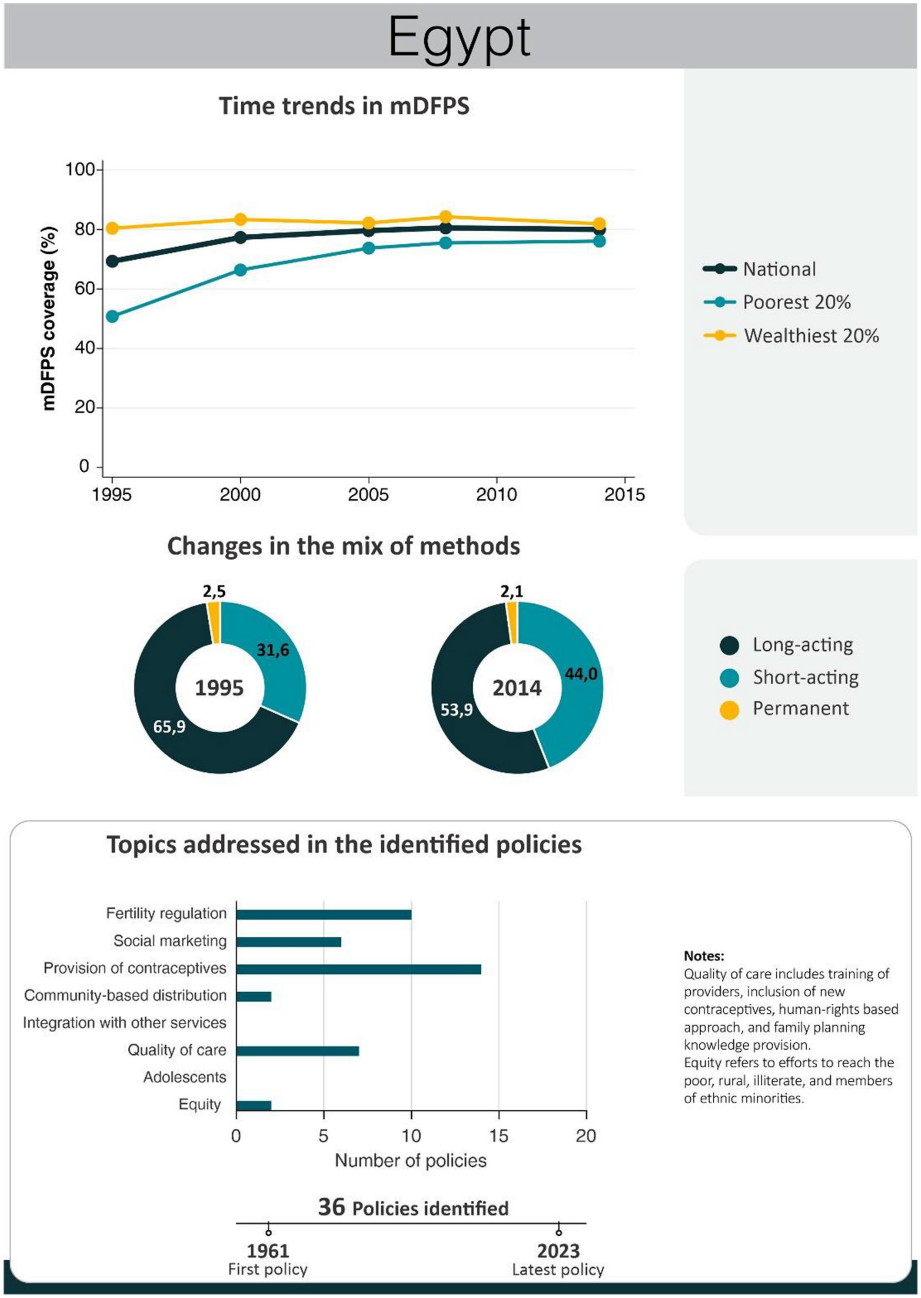
National trends in demand for family planning satisfied by modern methods and among the poorest and the wealthiest women, share of modern contraceptive use in the first and in the last national health survey, and topics addressed by policies implemented in Egypt. Source: Demographic and Health Survey.

**Figure 4:**
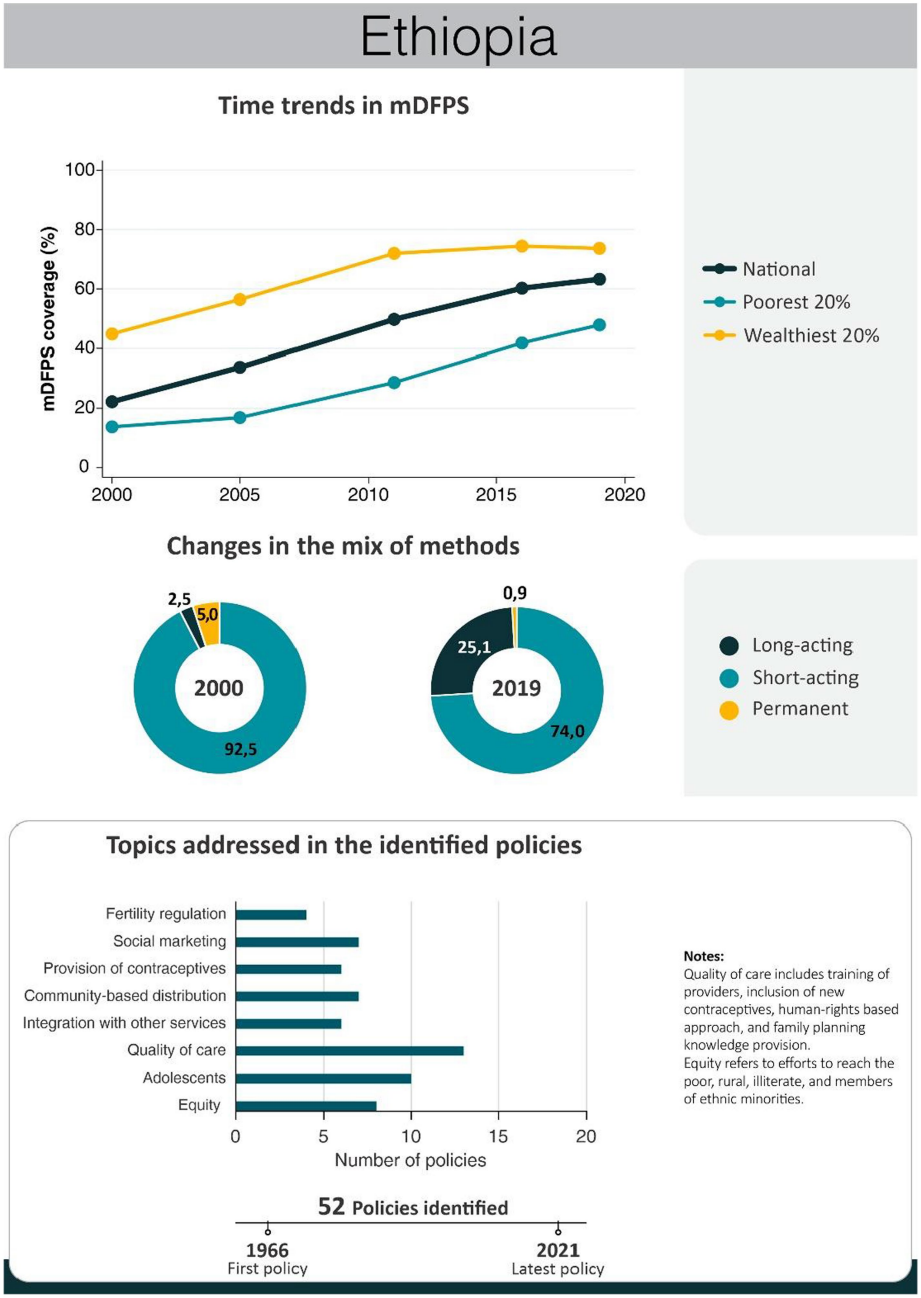
National trends in demand for family planning satisfied by modern methods and among the poorest and the wealthiest women, share of modern contraceptive use in the first and in the last national health survey, and topics addressed by policies implemented in Ethiopia. Source: Demographic and Health Survey.

**Figure 5:**
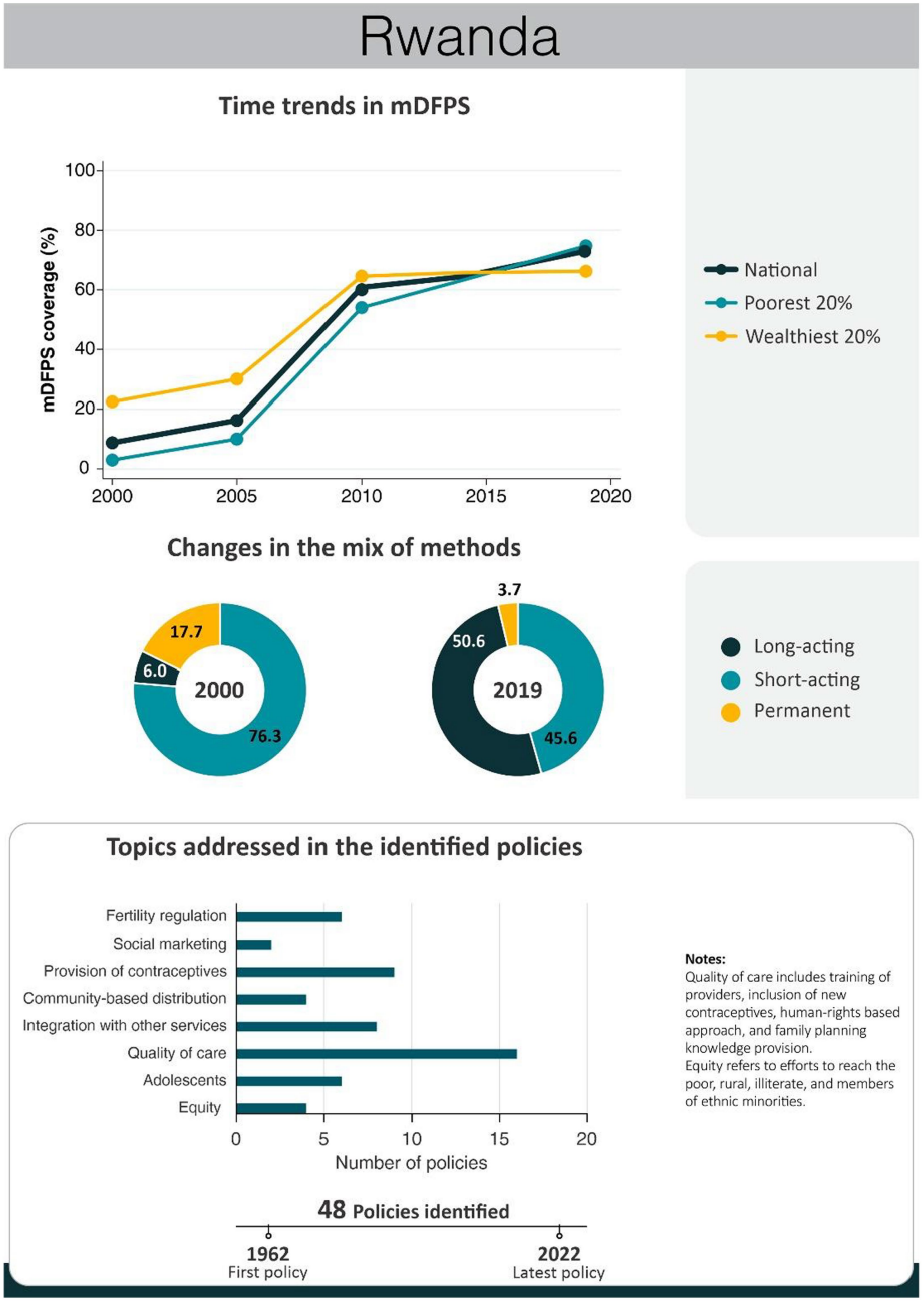
National trends in demand for family planning satisfied by modern methods and among the poorest and the wealthiest women, share of modern contraceptive use in the first and in the last national health survey, and topics addressed by policies implemented in Rwanda. Source: Demographic and Health Survey.

An overview of the improvements in family planning coverage within each country and the topics addressed by the identified policies is presented in Figures 1–5. All countries started to endorse family planning during the 1960s. The leading country in terms of the number of policies was Ethiopia, where 52 interventions have been implemented since 1966. We identified 48, 39, 36, and 21 policies in Rwanda, Brazil, Egypt, and Ecuador, respectively. A significant proportion of these policies were aimed at facilitating basic contraceptive provisions and enhancing the overall quality of family planning services. All countries enacted policies targeting the sexual and reproductive health of women across varying age groups whether married or unmarried. The exception was Egypt, where only married women were targeted. Many of these interventions lacked thorough evaluation, with neither the implementing organizations nor scientific studies adequately assessing their impact and effectiveness.

The subsequent sections explore the experiences of each country, organized alphabetically and by region.

### 3.1 Brazil

Between 1986 and 2019, mDFPS in Brazil increased from 80 to 93%. The richest women already had over 90% of demand satisfied in 1986 compared to just over 50% for the poorest women. That gap disappeared by 2019. At the same time, an important decrease in the share of permanent methods (nearly all female sterilization) was observed, from 60% in 1986 to 30% in 2019. Short-acting methods, concurrently, increased from 40 to 65% of all methods used. Long-acting methods had a modest increase, from 1 to 5% in the period (Figure 1). Most of the policies implemented in Brazil involved the provision of contraceptives, strategies to improve the quality of services, and to promote sexual and reproductive health services to adolescents (Figure 1). A timeline of the main policies and programs is presented in Table 1. A complete list of the identified policies is presented in Supplementary material.

**Table 1:** Timeline of the main policies for family planning in Brazil.

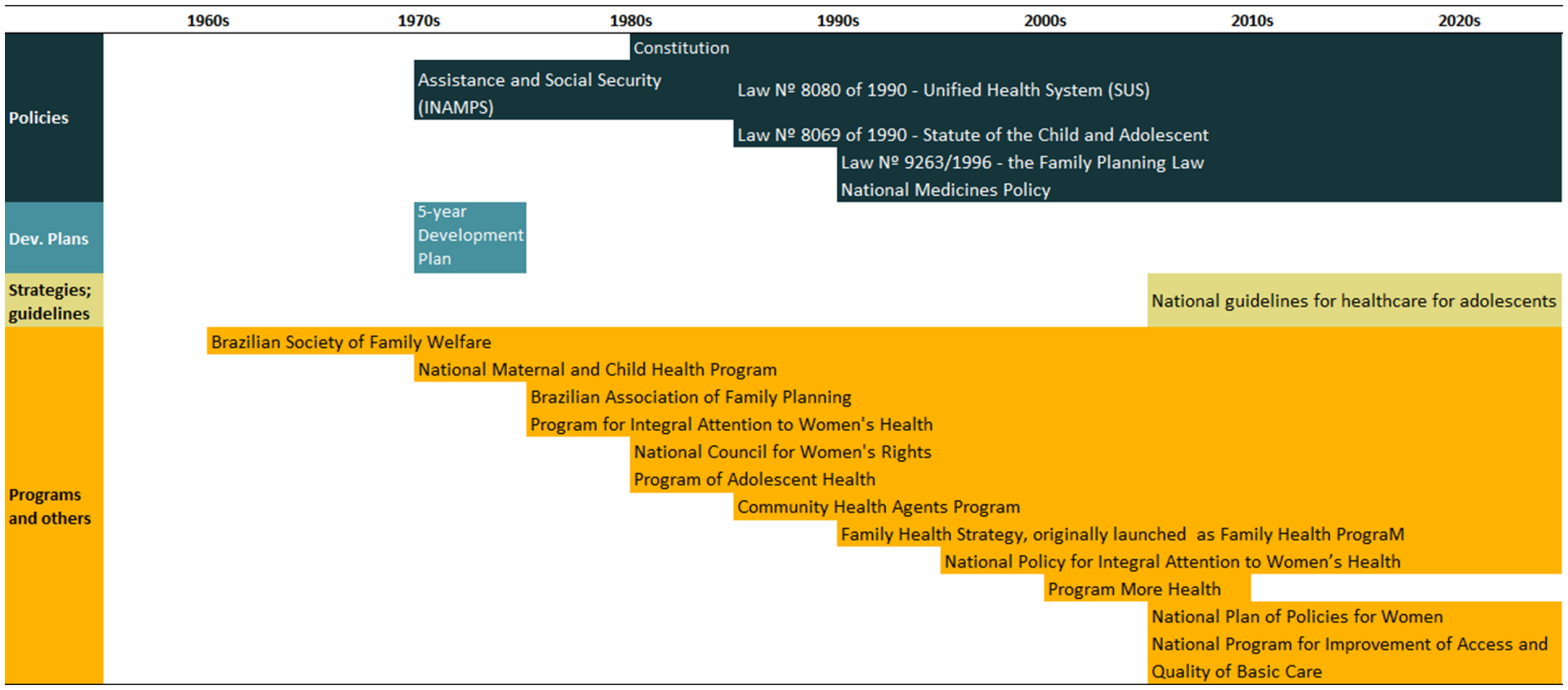

For nearly a decade, the Brazilian Society of Family Welfare (BEMFAM, original: *Sociedade Civil de Bem-Estar Familiar no Brasil*), established in 1965 and funded by international organizations, was the sole provider of family planning services in Brazil. Despite initial legal opposition to contraceptive use, BEMFAM devised strategies to foster family planning (29, 30). This encompassed setting up family planning centers, training courses for family planning providers, and deploying trained volunteers for method distribution in underserved regions (31–35). Other important institutions were the Brazilian Association for Family Planning Entities (ABEFP, *Associação Brasileira de Entidades de Planejamento Familiar*), which organized and promoted several private programs, including training courses and provision of equipment for consultation rooms (36), and the Centre for Research and Integral Care for Women and Children (CPAIMC, *Centro de Pesquisa e Atenção Integrada à Mulher e à Criança*), which trained doctors in family planning based on the tubal ligation (37).

The Brazilian health public policy during this period was the National Institute of Medical Assistance and Social Security (INAMPS, *Instituto Nacional de Assistência Médica da Previdência Social*), destined to formal sector workers. Confined to improve health conditions, in 1986 the government passed an act to provide family planning services, recognizing the human right to deliberate about reproductive life (38).

The initial programs with relevance to family planning in Brazil were the National Maternal and Child Health Program (PMI, *Programa Materno-Infantil*), launched in 1975, and the Program for Integrated Women’s Health Care (PAISM, *Programa de Assistência Integral à Saúde da Mulher*), initiated in 1983 and regulated in 1986. PMI often neglected family planning within its health package due to limited resources (35, 39). While PAISM included a family planning component, contraceptive methods, such as condoms, pills, and copper IUDs, were not provided regularly during its implementation (40, 41). Despite its shortcomings, PAISM was instrumental in advancing reproductive health (35, 41, 42). It is impossible to evaluate the impact of these programs on family planning since no statistics on modern contraceptive use were available up to 1986.

In 1985, the National Council for Women’s Rights was created under the Ministry of Justice. It was essential for the inclusion of women’s demands in the consultation process for the new Brazilian Constitution (43, 44). This council organized campaigns to promote women’s health, recognizing family planning as the mean to eliminate the biological risks of pregnancy. It also helped to overcome religious barriers related to modern contraceptive methods use, such as the promotion of the idea that not only fertility awareness methods could be used for family planning (44, 45).

Brazil’s 1988 Federal Constitution acknowledged health as a citizen’s right and a state responsibility (46), laying the groundwork for the Unified Health System (SUS, *Sistema Único de Saúde*). The SUS was based on universality (unrestricted health access), integrality (holistic care provision), and equity (equal care for all), and was established via law #8080 of 1990. Since then, Brazil’s healthcare, including family planning, has witnessed significant advancements in all areas (47).

A national program of community health workers was created in 1991 (PACS, *Programa de Agentes Comunitários de Saúde*). It aimed to hire and train health workers to interact directly with their communities specifically on disease prevention and health promotion actions (48). This initiative paved the way for the Family Health Program (PSF, *Programa Saúde da Família*), launched in 1994 (49). PSF was a tremendous success in the provision of primary healthcare services to the most vulnerable populations, being it designed to promote equity. Municipalities received funding and incentives to implement new PSF units in places where a health facility was not available. This was an important change from other programs where funds were mostly used in existing facilities, perpetuating a lack of service provision in more remote areas. Another key characteristic of the PSF was the idea of a health team, comprising a doctor, a nurse, auxiliary nurses, and community health workers. The program was subsequently expanded and renamed to Family Health Strategy (ESF, *Estratégia Saúde da Família*) (50), now present across Brazil. Although ESF’s focus is broader, evidence suggests it positively impacted contraceptive use, attributed to enhanced primary healthcare unit coverage (51).

Brazil’s drive to enhance adolescent health began with the 1989 Program of Adolescent Health (PROSAD, *Programa de Saúde do Adolescente*). In a context of high rates of adolescent pregnancy, one of the targets of the PROSAD was on contraception and prevention of sexually transmitted infections (52). The Statute of the Child and Adolescent, passed as law in 1990 (law 8.069/1990), allowed physicians to provide contraceptives to adolescents without the presence of parents or their consent, providing more autonomy to adolescents (34, 52, 53).

Although more sophisticated modern contraceptives were increasingly available in the 1990s, only the upper classes had access to a wider mix of methods. Poor women were mostly using female sterilization, performed with unnecessary cesarean sections (37, 54). Additionally, modern contraceptives were highly incorrectly used, leading to negative health effects, unwanted pregnancies, and illegal abortions (55). In 1996, a new law (9.263/1996), known as “the family planning law,” reinforced a holistic and comprehensive vision of health and stated that family planning was an integral part of health care. It also included regulations for the surgical sterilization (56, 57).

From 1998, investments, particularly in women’s health, expanded. These funds were mainly used to buy commodities related to family planning and to strengthen the technical knowledge and the actions promoted by states, municipalities, and non-governmental organizations (NGOs) (44). Concurrently, the National Medicines Policy (*Política Nacional de Medicamentos*, ordinance 3,916/1998) promoted drug rational use and included a list of essential medicines that should be made freely available through the healthcare network. The current list, RENAME 2022, includes male and female condoms, injectables, copper IUD, diaphragm, and oral contraceptive pills (58).

Directly related to sexual and reproductive health, the National Policy for Integral Attention to Women’s Health (PNAISM, *Política Nacional de Atenção Integral à Saúde da Mulher*), was approved in 2004 and reviewed in 2017. Regarding contraception, it aims at the expansion of family planning actions by providing contraceptive methods to women in need and improving information about them. Currently, according to the program, the following modern contraceptive methods should be available free of charge in public health facilities: condom (male and female); injectable (monthly and quarterly); copper IUD; combined contraceptive pill; minipill (progestin-only pill); emergency contraception (levonorgestrel-only pill); diaphragm; and sterilization (male, female) (33).

The 2004 National Pact for the Reduction of Maternal and Neonatal Death aimed to establish sexual and reproductive healthcare protocols, including contraception access, especially for adolescents (52, 59). Other initiatives targeting adolescents were launched in the following years. The 2005 Adolescent Health Program and the 2007 Theoretical and Reference Framework: Sexual Health and Reproductive Health of Adolescents and Young People, alongside the Health Legal Framework for adolescent rights, furnished health professional guidance and strategies for sexual education and service provision based on sexual and reproductive rights (52). Under the 2009 Adolescent Health Policy, the adolescent health handbook encompassed diverse topics, including sexual education, bodily development, sexually transmitted infections, contraception, and life planning (60). In 2010 the Ministry of Health introduced new guidelines for adolescent healthcare services, encompassing crucial aspects such as gender equity, youth participation, and sexual and reproductive rights. This document also emphasized the need for comprehensive policies that intersect for optimal outcomes (52, 61).

Until 2006, a significant gap existed between proposed measures and their actual implementation within health services. Beyond contraceptives’ unavailability in public health initiatives, family planning efforts often remained disjointed from broader health activities. Health professionals and managers frequently failed to perceive family planning as an integral part of primary healthcare (62, 63). Additional barriers encompassed provider training, healthcare management, and inadequate respect for women’s autonomy (63). Despite the gaps and weaknesses in the Brazilian sexual and reproductive policies, real progress has taken place, mainly because the agenda was sustained and expanded by civil society and academia. Women’s sexual and reproductive rights initiatives were also consolidated in the period (42).

The “More Health: everyone’s right 2008–2011” (*Mais Saúde: um direito de todos* 2008–2011) program included 86 goals and 208 actions to improve the healthcare system. On family planning, these actions focused on furnishing information and contraceptive methods under the 2007 National Policy of Family Planning. This policy, in addition to what was already available, introduced male sterilization in outpatient services and incorporated contraceptives into the Popular Pharmacy Program (39). These programs, a partnership between public facilities and private pharmacies, offered subsidized medicines to the population (40). Contraceptives were included in the program in 2008, with contraceptive pills being sold at 10% of their market price (39). In 2009, over 30 million women accessed free contraceptives through public health facilities and more than 10,000 retail pharmacies were participating in the Popular Pharmacy Program (39). Additionally, with easier access to male sterilization in outpatient clinics, the procedure had an increase of 83% between 2007 and 2009 (39). A Brazil-based study in 2013/2014 found that just 5% of out-of-pocket-paying contraceptive users availed themselves of the discounted prices offered by the Popular Pharmacy Program for oral contraceptives, with the rest purchasing them at full price from private pharmacies (64). Most of the out-of-pocket-paying users did not try to buy their methods through the Popular Pharmacy Program (64). This is a clear indication that despite the SUS offering free or cheap alternatives, the private sector has an important complementary participation in the provision of health services, offering a wide range of family planning options to people who are willing to pay out-of-pocket or through health insurance.

Launched in 2011, the National Program for Improvement of Access and Quality of Basic Care (PMAQ-AB) aimed to elevate healthcare service quality. Multiple strategies were deployed, encompassing service qualification, follow-up, and evaluation. Cities demonstrating higher-quality services were allocated greater financial resources. The program led to a surge in availability, rising from 1.5 to 10.9%, with male condoms and pills being the most accessible, and IUDs the least. The highest improvements were observed in low-development municipalities (65).

The 2013–2015 National Plan of Policies for Women (PNPM, *Plano Nacional de Políticas para as Mulheres*) aimed to reinforce the PNAISM, providing high-quality sexual and reproductive health services (43, 44). Other recent efforts were related to long-acting reversible methods, increasing the availability of copper IUDs in public maternity hospitals, and incentivizing their use in the immediate postpartum period (66). The postpartum IUD insertion was also reinforced in 2017 by the Stork Network (41, 42). The Stork Network, established in 2011, prioritized women and infant rights, emphasizing humanized care during pregnancy, childbirth, and the postpartum period, while reducing maternal and infant mortality within the SUS. Since its implementation, quality care metrics were introduced to monitor advancements.

Other efforts were made concerning the provision of long-acting reversible contraceptives. The Levonorgestrel-releasing intrauterine system (LNG-IUS) Program of the International Contraceptive Access Foundation, which has been distributing IUDs to health centers (67), and the Normative Resolution No. 167 of 2008 of the National Agency of Supplementary Health, which implemented a requirement for health insurance plans to offer IUDs as part of their coverage (68). Despite all these efforts, long-acting reversible methods still have a very limited participation in the mix of modern methods used in Brazil.

### 3.2 Ecuador

Our previous analyses revealed that mDFPS presented a steep increase in Ecuador from 1986 to 2012 (27). Using survey data, we see that from 1994 to 2018, Ecuador not only had an overall increase in mDFPS from 67 to 87% but that wealth inequalities disappeared. The share of methods also changed, with an increase in short-acting methods and a decrease in long-acting methods. Ecuador’s policies chiefly centered on healthcare service quality and adolescent outreach (Figure 2). A timeline of the main policies and programs implemented in Ecuador is presented in Table 2. A complete list of the identified policies is presented in Supplementary material.

**Table 2:** Timeline of the main policies for family planning in Ecuador.

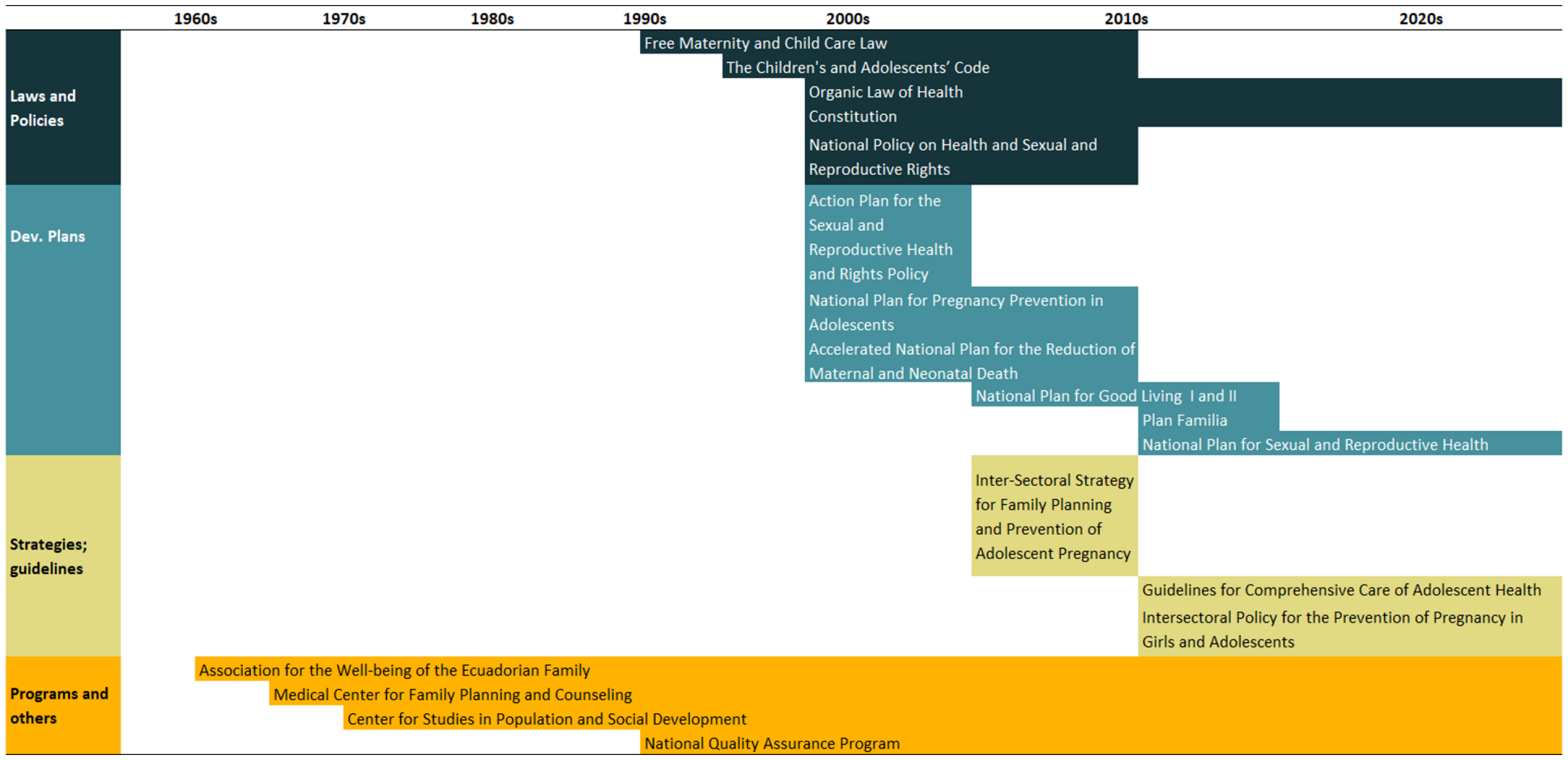

The United States Agency for International Development (USAID) and the International Planned Parenthood Foundation (IPPF) were donors who made important contributions to Ecuadorian reproductive health, supporting key institutions (69). Both supported the Association for the Well-being of the Ecuadorian Family (APROFE, *Asociación Pro Bienestar de ka Familia Ecuatoriana*) and the Medical Center for Family Planning and Counseling (CEMOPLAF, *Centro Médico de Orientación y Planificación Familiar*), institutions that have facilitated family planning progress by bolstering modern contraceptive provision—particularly IUDs—, enhancing reproductive health services quality, and advocating for women’s rights (70).

Founded in 1965, APROFE pioneered private voluntary reproductive health efforts, aiming to uplift income generation and accessibility for the underprivileged (71). The private non-profit organization CEMOPLAF, launched in 1974, focused on equitable sexual and reproductive health access, prioritizing disadvantaged groups, especially adolescents and poor women (72). The Center for Studies in Population and Social Development (CEPAR, *Centro de Estudios de Población y Paternidad Responsable*), which started in 1978, was another relevant initiative in Ecuador, focusing on educational activities about population growth and family planning issues, promoting family planning in different settings, publicly and politically (73).

In 1974, Ecuador’s government took direct steps toward supporting family planning by announcing a comprehensive plan to extend these services to all citizens. Family planning was integrated into public health services as a medical initiative to improve maternal and child health, with information and education being recognized as important aspects of family planning services. Oversight and management of both public and private family planning services were centralized under the Ministry of Health (74). While aiming for standardized services, this central control posed obstacles for international organizations providing support to family planning programs in Ecuador (75).

The Free Maternity and Child Care Law was a family planning milestone. This law was passed in 1994 and updated in 2006, granting women access to sexual and reproductive health programs spanning prenatal, childbirth, and postpartum care. This law guaranteed that the provision of at least 72 health benefits would become freely available for women at reproductive age, including access to modern contraceptives (76). In addition, it created an autonomous executive unit attached to the Ministry of Public Health with the responsibility to ensure the proper implementation of the Law and the correct use of the allocated resources. Another entity that was created by the law was local user committees to promote citizen co-responsibility in maternal and child health, guaranteeing that this law was put into practice. The budget to finance this law was established at US$ 15 million per year and 3% of special consumption taxes, corresponding to around US$5 million, guaranteeing US$ 5 million exclusively for the acquisition of modern contraceptive methods (77).

In 2006, the Organic Law of Health was sanctioned and later regulated in 2012. It set overarching guidelines for family planning initiatives, emphasizing respect for women’s and men’s rights. In consonance with this law, complementary endeavors fortified family planning efforts. The National Policy on Health and Sexual and Reproductive Rights emerged in 2007, favoring human rights, gender equality, and maternal mortality reduction. It also proposed health system reforms aiming to promote access for all (78). The Action Plan for the Sexual and Reproductive Health and Rights Policy (2006–2008) prescribed various actions aligned with the National Policy, advocating rights, dismantling access barriers, promoting autonomy in sexual and reproductive matters, and prioritizing problems such as maternal mortality, abortion, and unwanted pregnancy (78).

Ecuador implemented several policies to enhance sexual and reproductive health services for adolescents. The 2003 Children’s and Adolescents’ Code underscored adolescents’ health rights, mandating free high-quality health services through the national health system (79). Additionally, the 2007 National Plan for Pregnancy Prevention in Adolescents prioritized gender equality and pregnancy reduction (80). This plan revolved around bolstering health, education, and social protection, and adopting an interinstitutional approach with social involvement (79, 81, 82). Also in 2007, specialized services for adolescents were implemented. These services included trained staff in dealing with adolescents, facilities with a friendly atmosphere, respect for adolescents’ privacy, and confidentiality (83). In 2009, the New Guidelines for Comprehensive Care of Adolescent Health were launched, advocating youth-friendly services nationwide (79). Ecuador initiated the Inter-Sectoral Strategy for Family Planning and Prevention of Adolescent Pregnancy (ENIPLA) in 2011 (84). This policy was a key step in the prevention of adolescent pregnancy in Ecuador, adopting a comprehensive family planning approach. Beyond contraceptive access, it supported women and their families to achieve fundamental rights such as a life free of gender-based violence, and also contributing to cultural changes (84–86). ENIPLA was an intersectoral strategy based on interministerial collaboration, focused on four main goals: 1. keeping adolescents in the educational system and strengthening comprehensive sexual education; 2. enhancing adolescent access to sexual and reproductive health services, including contraceptive methods; 3. Nurturing family and community dialogue; and 4. promoting changes in socio-cultural norms (83, 87, 88).

The use of modern contraceptives increased progressively, especially from 2008, due to the increase in social spending and health policies introduced at the time of the reform, which improved the performance of primary health care services (89, 90). The adolescent fertility rate was reduced from 80 births per 1,000 women in 2007 to 71 in 2017 (91). The 2008 Accelerated National Plan for the Reduction of Maternal and Neonatal Death, a priority public policy for the health sector, delineated actions to amplify healthcare quality and services throughout Ecuador (78, 81). In the same year, the Constitution caused a change in the concept of health access as a right, mentioning fundamental sexual and reproductive rights, such as the freedom to make decisions about health and reproductive life and to decide how many children to have. Equality and nondiscrimination are other rights guaranteed in the constitution, along with the State’s responsibility to ensure reproductive health, offering information and support for women to make decisions on family planning.

As a result of the constitution, a reform in the National Health System was made and a new model for integrated family and community health care was launched. The Model of Comprehensive Family, Community, and Intercultural Health Care focused on integrated care throughout the lifecycle and on family and community medicine, taking the focus off units specialized for specific subgroups (83, 84). As a result, vertical programs, such as specialized services for adolescents, were abolished (92).

Although Ecuador’s Constitution defined health as a universal right and determined free healthcare, the Free Maternity and Child Care Law was dismantled as well as its executing unit. In 2014 the ENIPLA also was closed through a presidential decree, weakening the management of sexual and reproductive health at the national level (93). Other notable events include the weakening of Assured Availability of Contraceptive Methods and Supplies (DAIA), diverting the earmarked US$ 5 million for contraceptives into the Ministry of Public Health’s general expenses, and jeopardizing consistent investments in family planning.

Replacing ENIPLA, the National Family Strengthening Plan Project was introduced in 2015. The plan aimed to “reclaim the leading role of the family and contribute to the development of all dimensions of the human being in the lives of adolescents.” (93, 94). Executed by the Ministries of Education, Public Health, and Economic and Social Inclusion, this initiative received over US$ 24 million for implementation between 2015 and 2017 (94). This Plan represented a change of approach in Ecuador, with more restrictive access to sexual and reproductive health services and possible impacts on the adolescent birth rates (93, 94). The Plan prioritized family-based sexual education to delay sexual activity initiation and encourage abstinence. It was also critical of some aspects of previous actions adopted in this country (93, 94). In 2017, this plan was nullified nationally through a presidential decree.

The National Plan for Good Living promoted actions, implemented from 2013 to 2017, outlining 12 goals across six dimensions to enhance population well-being. Ensuring access to sexual and reproductive health services was a targeted objective (85). The 2017–2021 edition of the plan updated the previous version by establishing three thematic axes (universal rights throughout life, economy at the service of the society, enhanced social participation and state actions) (85). Within this framework, sexual and reproductive rights play a pivotal role in well-being.

The National Plan for Sexual and Reproductive Health 2017–2021 was also launched in 2017. This plan established clear guidelines for sexual and reproductive health, defining annual targets as well as the necessary budget to achieve them (95). Among its strategies was reinforcing family planning services through funding for modern contraceptive methods (95). In 2018, Ecuador resumed the implementation of rights-based gender-focused policies for the prevention of pregnancy in girls and adolescents through the Intersectoral Policy for the Prevention of Pregnancy in Girls and Adolescents (2018–2025). This policy places shared responsibility for adolescent pregnancy on the sectors of health, education, socioeconomic inclusion, and justice. One of the strategic lines of this policy is dedicated to ensuring Comprehensive Sexual Education, the exercise of sexual and reproductive rights, adolescent-friendly healthcare services that provide evidence-based information, and effective access to modern contraceptives without discrimination based on age, ethnicity, migratory status, among other factors (70).

Currently, the array of available modern contraceptive options encompasses condoms, pills, emergency contraception (pills), IUDs, implants, injectables, and sterilization (77, 95). Through pharmacies and private clinics, the private sector also plays an important role, being the source of 38% of modern contraceptives used (95). Until 2018, contraceptives were readily accessible at no cost in public healthcare facilities (95). However, a reduction in the budget allocated to the Ministry of Public Health led to cutbacks in relevant projects. This underscores the need for the country to secure annual resources for procuring modern contraceptive methods, intending to address the persistent unmet family planning needs.

### 3.3 Egypt

In Egypt, modern contraceptive use increased from 19 to 57% between 1976 and 2014. The fastest increase occurred pre-2000, maintaining a contraceptive prevalence of 50–60% thereafter. Demand for family planning satisfied by modern methods is currently at 80%, with an important reduction in wealth inequalities since 1995 mainly due to a steeper increase among the poorest women. The distribution of contraceptive methods did not change significantly in the 1995–2014 period. Permanent contraception accounts for a minor fraction, with long-acting methods predominating. Egypt’s endeavors chiefly concentrated on modern contraceptive provision and advocating for smaller families, leveraging robust social marketing campaigns (Figure 3). A timeline of main Egypt’s policies and programs is presented in Table 3, while a complete list of the identified policies is presented in Supplementary material.

**Table 3:** Timeline of the main policies for family planning in Egypt.

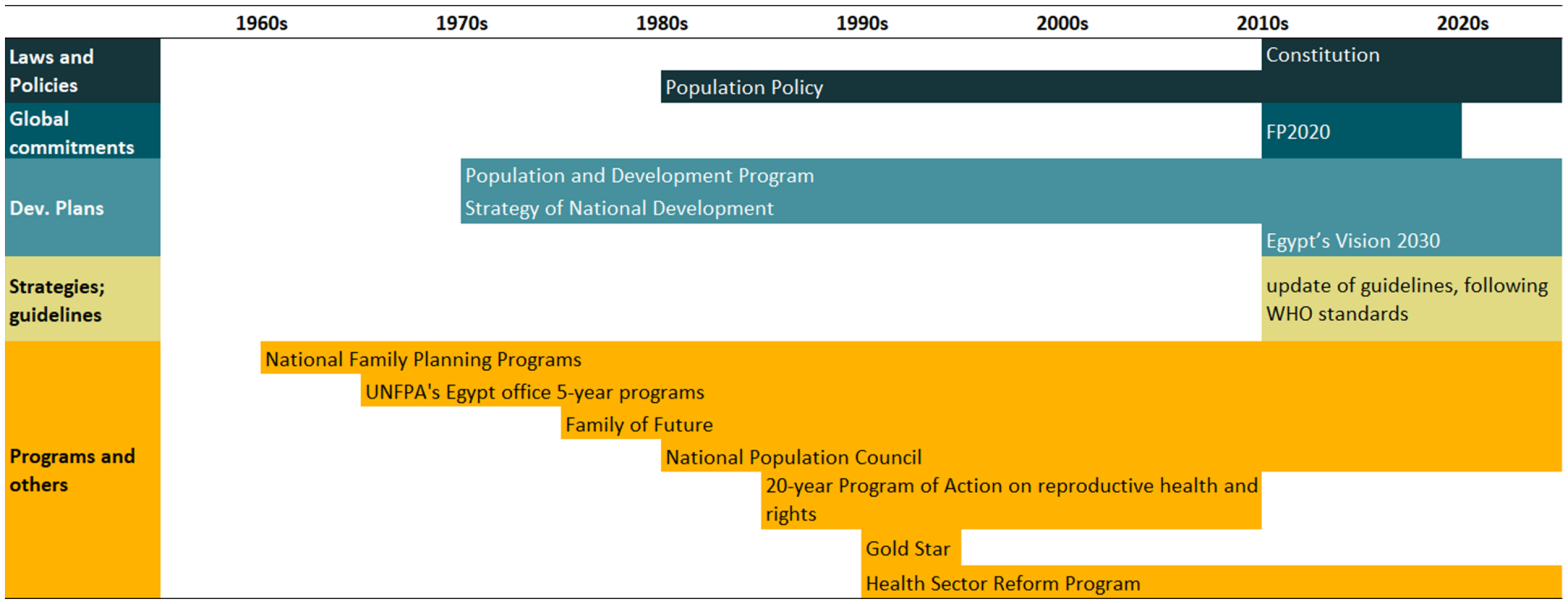

Contraceptive availability in Egypt expanded during the 1950s through women’s private voluntary associations advocating social services, healthcare centers, and international collaborations (96). On a different front, the state began to endorse family planning on the 1962 Charter for National Action and the following national family planning program, implemented in 1966 (96–98). These early endeavors aimed at fertility reduction, creating health clinics, and promoting modern contraceptives (4, 97–99). In this context, international organizations played a crucial role, through funding, the provision of technical assistance, training of health providers, and contraceptive commodities. Important organizations were the World Bank, the Ford Foundation, the Population Council, the Pathfinder Fund, and the USAID (96, 97). However, some societal groups perceived these initial efforts as an attack on women’s autonomy and ability to make informed fertility decisions, given that the program was implemented focusing on having many facilities and providing certain types of contraceptives rather than advancing women’s health and population control by education (97, 98).

In the following years, several organizations were created aiming to increase and improve access to family planning among Egyptian women. The Egyptian Family Planning Association, the Joint Committee for Family Planning, and the Supreme Council for Family Planning (96, 100). UNFPA’s Egypt office since 1972 backed family planning through eleven 5-year programs (101–103).

Through a developmental approach to Egypt’s population program and looking for integrated family planning and development efforts, Egypt launched in 1977 the Population and Development Program (PDP) (99, 104, 105). The program aimed to accelerate economic development, slow population growth, and improve community health and welfare. It stimulated family planning through a system of semi-volunteer outreach workers and infused family planning messages in other services (99, 104, 105). Evaluation of the program has shown that it yielded positive impacts on contraceptive knowledge, attitudes, and practices (104, 106).

Following the 1979 Convention of the Elimination of all Forms of Discrimination against Women, Egypt launched its most significant program related to reproductive health, the Family Planning Program, as part of its reproductive health agenda. The increase in modern contraceptive use during the initial decades was bolstered by a massive increase in the number of service delivery outlets (107). The number of pharmacies, the main providers of pills and condoms, increased eight times between 1978 and 2004 (107). During this period, the financial resources allocated to family planning increased 5 times in nominal terms. The biggest share of this investment was covered by the national government, followed by international donors and NGOs. The out-of-pocket expenditure in family planning also increased about five times (107). Counterfactual analyses indicated that contraceptive use would have increased much less without the program (107).

Alongside the national program, the Family of the Future (FOF), a private family planning organization supported by the IPPF and the USAID, promoted family planning via media campaigns and physician training, with a focus on IUD insertion (108, 109). The Information, Education, and Communication (IEC) Center, established in 1979, bolstered family planning through several mass media campaigns and community-based public communication activities (110, 111). In a context of a high level of illiteracy, television campaigns proved highly effective in disseminating family planning information, with a massive increase in contraceptive use observed after their implementation (110, 112). Family planning was also a component of socioeconomic development plans implemented in the period (99, 113).

In the 1980s, the National Population Council was established, to reduce birth rates and elevate contraceptive prevalence (114). Concurrently, a fresh family planning program and population policy were implemented, both geared toward curtailing population growth (115). During this phase, Egyptian policymakers held a strong conviction that controlling the fertility rate would drive economic development, rather than the reverse (116). In this period population matters were incorporated into educational curricula and guidelines were provided to doctors on how to convince families to adopt family planning practices (114, 116). Television propaganda underwent sophisticated enhancements, featuring intricate narratives, and renowned actors portraying rural characters (117), which was the population subgroup more reluctant toward state-provided family planning services (98).

Starting in 1992, Egyptian family planning services expanded their focus to encompass service quality. The Ministry of Health initiated a campaign aimed at enhancing client satisfaction with family planning services, employing a comprehensive checklist of over a hundred indicators, including method availability and facility conditions. Clinics deemed of high quality were marked with a distinctive seal to facilitate customer recognition (118). In 1995 the government initiated the Gold Star program, effective until 2000, to enhance family planning quality and utilization. It notably expanded the availability of methods, enriched counseling quality, and improved examination services (119). Another impactful approach was the 1997 Health Sector Reform Program, which aimed at improving how primary health care was financed, managed, and delivered. It standardized family planning services via an accreditation system (120–122). Although the effect of the accreditation process was not very strong, accredited facilities showed higher contraceptive usage, informed clients about contraceptive side effects, and boasted superior service satisfaction compared to non-accredited facilities (120, 121).

Following the 1994 Cairo International Conference on Population and Development, Egypt adopted the 20-year Program of Action on reproductive health and rights. This program aimed to enable couples to make informed decisions about family size and spacing through accessible information and means. It emphasized diverse contraceptive choices to suit women’s needs, advocating for male participation and shared responsibility in family planning (123).

These initial policies sparked both a rapid surge in contraceptive prevalence until 2000 and a notable uptake of long-acting reversible methods. The social marketing strategies and the grassroots women’s advocacy appeared pivotal in stimulating demand, facilitated by the increase of family planning clinics. By the early 2010s, with the investments made in health care, the quality of the services had improved and 95% of the population was living no more than 5 km away from a health facility, with all sites offering contraceptives. The state provided oral pills at subsidized rates, while NGOs took the lead in furnishing vaginal foam tablets, pills, and IUDs (96). The large share of long-acting reversible methods was also achieved by the training of doctors to insert IUDs (96, 100).

The Egypt Ministry of Health and USAID launched in 2003 the Communication for Health Living project, which included a campaign focused on family planning. “Your Health, Your Wealth” was a nationwide television campaign focused on birth spacing benefits. The evaluations indicate positive effects of the campaign on spousal discussion about family planning and modern contraceptive use (124).

A more recent development strategy is the Egypt’s Vision 2030, launched in 2016. The strategy aimed to improve the quality of healthcare services and their provision and management in general. One of its targets is specifically related to family planning coverage, aiming to increase the ratio of women using new contraceptive methods (125). A subsequent important step occurred in 2018, involving the alignment of health professional training and family planning counseling guidelines with WHO standards. To bolster equitable access, all modern contraceptives were extended free of charge to the poorest villages, complemented by mobile clinics offering comprehensive no-cost services (126).

Egypt renewed its responsibility to family planning with its commitment to the FP2020 initiative, in 2017. In the following year, the government proposed family planning policies developed to strengthen the national commodity supply chain, improve the quality of human resources and services, and provide training courses on family planning. In addition, a specific system of accreditation for family planning clinics was established and high-quality clinics started to be recognized. The government also committed to continue increasing the national budget allocated to family planning and to develop new strategies to identify and deal with future needs (126).

In 2022, Egypt launched a new initiative, targeting family support and development. Family planning policies and programs, as well as gender equity, are fully embraced in this initiative. Aiming to support and encourage smaller families, this initiative started to offer life insurance and material benefits for families who only have two children, when the mother reaches age 50.

Acknowledging the importance of evidence-based decisions, the country developed an online repository named NPC POPLINE. This repository serves as a tool for informed decision-making in Egypt. When it was launched, in 2015, the database searched the studies on population and reproductive health in Egypt between 2005 and 2015. The repository is updated annually (127, 128).

While Egypt’s focal endeavors revolved around development and fertility reduction, some policies were targeted at specific subgroups, encompassing the poorest, illiterate, and rural women (129). Religious leaders have also contributed through educational programs and discourses (130–132). While these efforts contributed to low levels of inequality in terms of socioeconomic characteristics, family planning services are not yet readily available and affordable to young women, even married ones (133). Qualitative data indicate that adolescents and young women still face several legislative and cultural barriers when trying to access sexual and reproductive health services. Most of the young married women did not seek out these services before having their first child due to social expectations. To use public services, adolescents need to have marriage certificates or be accompanied by their mother or mother-in-law. However, most married adolescents do not have marriage certificates as marriages under 18 cannot be registered. Their families, on the other hand, will first instruct them to have a child (134). Additionally, young early married women are not expected to seek long-acting contraceptives due to fertility expectations (134). While many married young women end up looking for family planning in private services, their costs are substantially higher and seen as an important barrier (134).

### 3.4 Ethiopia

Over the past two decades, Ethiopia has achieved significant progress in family planning provision. mDFPS increased from 22% in 2000 to 63% in 2019. The gap in mDFPS between the poorest and richest women, however, did not change much. Along with the increase in demand satisfied, there was an important change in the method mix with the proportion of women relying on long-acting methods increasing considerably, from 3% in 2000 to 25% in 2019. Notably, the enhancement of family planning service quality and strategies catering to adolescent needs emerged as predominant themes in the policies implemented (Figure 4). Key policies and programs implemented in Ethiopia are presented in Table 4. A complete list of the identified policies is presented in Supplementary material.

**Table 4:** Timeline of the main policies for family planning in Ethiopia.

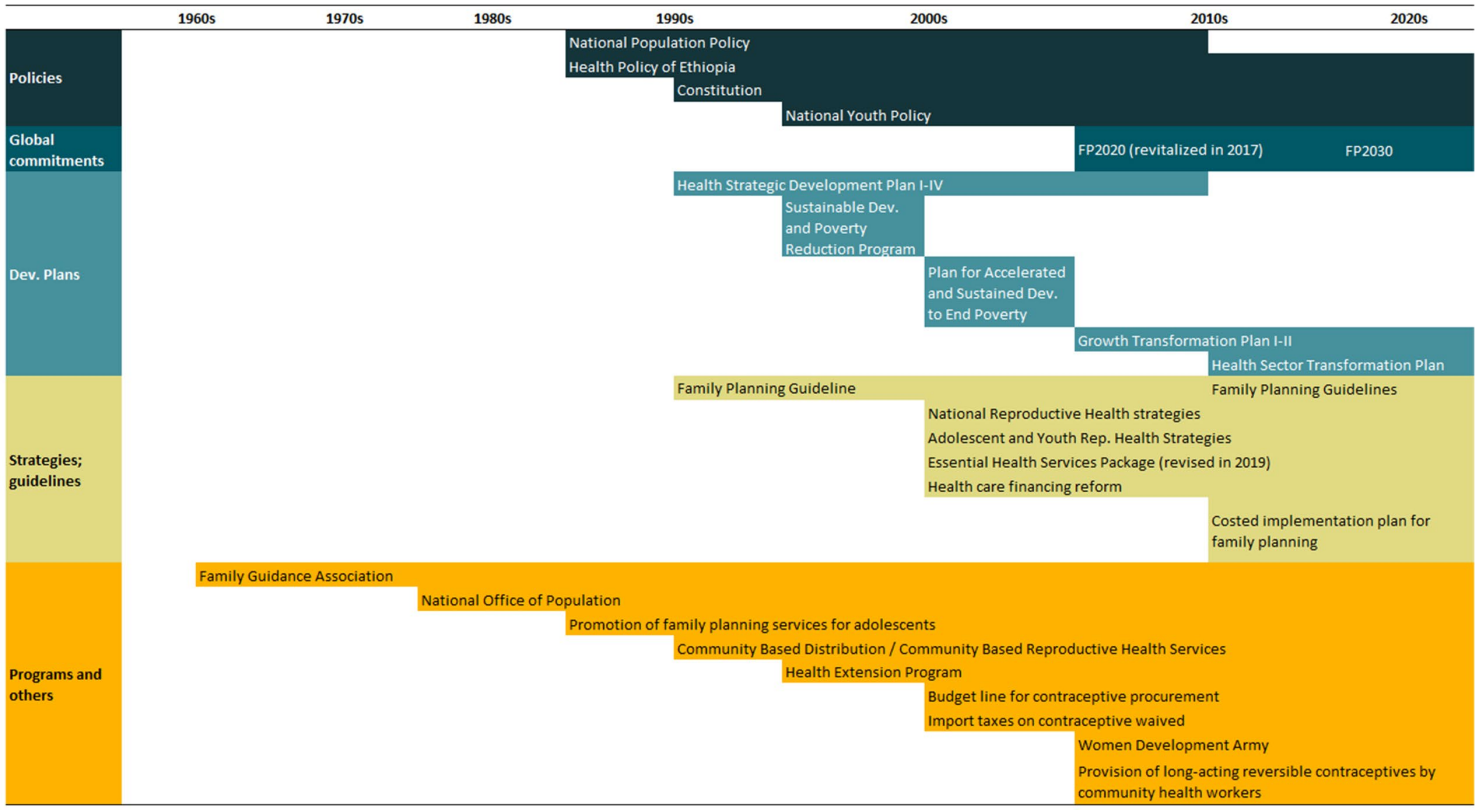

The improvement in family planning coverage over the past three decades in Ethiopia is a result of policies, strategies, and programs designed and implemented. The trajectory toward amplified family planning engagement traces back to the 1960s, notably with the establishment of the Family Guidance Association, linked to IPPF, in 1966 (135). In 1980, the Ministry of Health embarked on expanding family planning services with UNFPA and donor support. In the following year, the National Office of Population was established to develop and implement a national population policy. However, it was the democratic transition of 1992 that led to pivotal shifts in Ethiopia’s reproductive health policies. The first Ethiopian population policy, implemented shortly after the democratic transition, was implemented in the following, leading to significant increases in family planning coverage (135, 136). This policy acknowledged the correlation between chronic resource scarcities and population growth, and it aimed to align population growth with the country’s capacity, nurturing improved well-being conditions. It targeted curbing fertility rates and bolstering contraceptive prevalence, channeling resources from public and private sectors to expand family planning delivery through clinical and community-based channels. Other priorities were population research, education, provider training, social mobilization, and navigating legal constraints linked to contraceptive provision (136, 137). This marked the juncture where legal and customary opposition to family planning gradually waned, leading governmental and non-governmental health facilities to furnish family planning services (138).

A review of the implementation of the national population policy showed that it had improved knowledge and use of contraceptives by women. This progress emerged primarily from expanding family planning service facilities and diversifying service delivery approaches. The policy created a favorable environment to increase national and international partnerships for family planning service provision. It facilitated diverse strategies such as community-based reproductive health services, social marketing, mobile clinics, and social franchising to enhance awareness and accessibility of family planning and reproductive health services (89).

In 1993, Ethiopia also formulated a health policy intending to ensure the provision of comprehensive and integrated primary health care in a decentralized and equitable manner. The policy prioritized family health, particularly women and children. It emphasized preventive and promotive healthcare components and decentralization of health services. Within the policy, family planning services were identified as one of the strategies to promote optimal family health (136, 137).

In the early 1990s family planning services also started to be promoted for adolescents (139–142). The Youth Counseling Services and Family Planning Education Project and the Saturday Adolescent Family Planning/Counseling and Contraceptive Service established in 1990 and 1995, respectively, by the Family Guidance Association were important advances. These initiatives offered not just contraceptives, but also counseling and sexual education, disseminated through consultations, films, and radio dramas (141, 142). Services are a joint effort of staff, community, school teachers, and youth that aims to break down cultural barriers hindering service uptake (141).

An important driver for improved quality of family planning services was the African Regional Workshop organized by the IPPF in 1992. The workshop aimed to increase awareness and commitment to the quality of family planning services. Participants in this central workshop conducted following regional workshops that have fortified regional expertise in providing high-quality family planning services and in mapping out priority areas for intervention (143). Studies indicate that attendance has increased by 15–20% over the period and that client satisfaction also increased. Additionally, the promotion of family planning through mass media was further developed during this period (143).

The 1994 Constitution of Ethiopia affirmed equal rights to men and women and promoted the right of access to family planning education, information, and capacity (144). Article 35 specifically advocates women’s rights, with sub-article 9 underscoring their access to family planning education, information, and capacity to avert pregnancy and childbirth-related risks and safeguard health (144).

Based on a partnership between the German and the Ethiopian governments, the Community-Based Distribution program, a focusing on community-based services was implemented in 1995 (145). The program aimed to promote services to improve family health and family planning among hard-to-reach rural communities. Evaluations of the program showed positive results in knowledge about contraception and contraceptive use (145–148). The program was later expanded and renamed to Community-Based Reproductive Health Services (145). Also in 1995, the Essential Services for Health in Ethiopia (ESHE) Project was implemented. The first phase of the project was between 1995 and 2002, intending to increase the use of primary health care, including strengthening family planning (149). Strategies encompassed heightened contraceptive access, amplified social marketing for condoms and oral contraceptives, and promotion of training by international organizations (149). The second phase, 2003–2008, targeted health systems fortification, health worker efficacy improvement, and community engagement, with voluntary community health workers facilitating family planning provision (150). The ESHE presented considerable success in the delivery of family planning services, especially through NGOs (149).

Since 1996, Ethiopia has developed different guidelines and strategies for reproductive, maternal, neonatal, and child health services. The country developed the first family planning guideline in 1996, which was later revised in 2011 and then in 2020. The guideline of 1996 authorized new facilities to provide family planning and helped to expand and ensure the quality of services provided (151). With its implementation, all government health facilities extended contraception availability, introducing community-based services while NGOs and the private sector also joined in offering family planning services (138, 151). Government support also encouraged international donors to support family planning provision in the country (135). During this period, contraceptive use increased from 3% in 1990 to about 15% in 2005 (138).

The health sector of Ethiopia has also developed and implemented long-term health-sector strategic plans for the last three decades. Four rounds of Health Sector Development Plans (HSDP I to HSDP IV) have been developed and implemented from 1997/98 to 2014/15. Throughout these HSDPs, reproductive health services consistently assumed priority status. In the HSDP II (2002–2004), a new community-based program focusing on rural areas, the Health Extension Program (HEP), was introduced as a pilot. During the period, focus was given to the expansion of maternal health services and family planning by integrating them with other health services (152). The HEP was later expanded with family planning services being one of the main packages (153).

The HEP is one of the major platforms for the delivery of high-impact health promotion, disease prevention, and selected curative services to the community. With a primary aim of achieving universal health coverage, it delivers health promotion, prevention, and primary curative care, including family planning, to individuals living in the most neglected areas of the country. Through HEP, family planning services began to be delivered in the more vulnerable settings by female health extension workers (HEWs) working in health posts, outreach campaigns, and home-to-home visits (135, 154, 155). Initially, HEWs provided oral contraceptive pills, injectables, and condoms, but to increase access to long-acting contraceptives in rural areas, in 2009, HEWs started providing implant insertion. The implant scale-up program provided through the HEP was found to be a successful model for increasing access to long-acting reversible contraceptives in the community (156). In 2009, midwives were also allowed to provide IUD insertion services at health centers. In 2016, the Ministry of Health piloted a program (and scaled up in 2017) that trained level IV HEWs to insert and remove IUDs, insert and remove 2-rod implants, and remove one-rod implants. To ensure access and high-quality services for the urban community, the HEP was expanded to urban areas in 2009 (157, 158). Evaluation studies showed higher use of family planning services, including among adolescents, among urban HEP users in comparison with the standard services (159).

Economic evaluation has indicated that the HEP was very cost-effective and that it had been responsible for a substantial increase in family planning coverage (from 24 to 35% between 2005 and 2016) (154). In 2018, more than 97% of health posts were providing FP service (160), and HEP was the source of family planning information for 62% of Ethiopian women and 57% of current contraceptive users received it from HEWs (161). During this period, international organizations supported the Ministry of Health to introduce long-acting reversible methods in health centers across the country (162). The impact of these initiatives is visible from survey estimates that show the share of long-acting contraceptives to be 28% in 2016, from only 3% in 2000 (Figure 4).

The Women’s Development Army (WDA), a network of neighboring women, became part of the HEP in 2011, increasing the efficiency of HEWs in reaching every household. Through the strategy, women in the community, in partnership with HEWs, share and learn about health practices and empower one another. The implementation of the WDA strategy was found to be associated with better uptake of maternal health services, such as family planning services (163).

The national youth policies of 2004, 2007, and 2016 established an enabling environment for adolescents to access sexual and reproductive health information, education, and counseling services (164). However, despite these policies, adolescents and youth continued to suffer from elevated morbidity and mortality stemming from reproductive health implications (164).

Ethiopia’s commitment to the Millennium Development Goals (MDGs) played an important role in the substantial advancements witnessed in maternal and child health as well as family planning during the past two decades (102). The launch of the Plan for Accelerated and Sustained Development to End Poverty (PASDEP) in 2005 was a key contributor to these achievements (138). As a signatory to the Sustainable Development Goals (SDGs) in 2015, Ethiopia embarked on a strategic formulation to meet these objectives (165).

In 2006, Ethiopia introduced the Reproductive Health Strategy (2006–2015) to foster acceptance, demand, and access to quality family planning services. This strategy aimed to extend reproductive services to the lowest health tier possible without compromising safety or quality. Subsequent government actions included the elimination of import taxes on contraceptives and the promotion of intensive advocacy campaigns (135). Notably, the national health budget doubled between 2008 and 2013, augmenting the funds allocated for contraceptives provided by the public sector (135). The subsequent Reproductive Health Strategy (2016–2020) emphasized family planning improvement as one of its 12 strategic objectives, spotlighting contraceptive availability at all service delivery points, encompassing private health facilities, and expanding long-acting and permanent family planning methods (135). The recently designed third Reproductive Health Strategy (2021–2025) centers on quality and equity enhancement, along with an expanded reach of reproductive health services to workplaces, private facilities, universities, and humanitarian settings (166, 167). To cater to adolescent needs, user-friendly services were formulated through specific strategies initiated in 2007.

The Plan of Action for the 2008–2009 to 2015–2016 period aimed to strengthen National Population Policy implementation. Its objectives included integrating population concerns with development efforts, creating regional population action plans, and establishing efficient monitoring and evaluation mechanisms. Key actions included reducing unmet needs and increasing demand for family planning through information, education, and communication (136).

A study of the Private Health Sector Program found that, by 2015, despite the private sector’s potential in delivering family planning services, only 53% of private facilities were offering family planning services, and the quality of the services needs to be improved. To enhance private sector involvement in reproductive, maternal, neonatal, and child health services, and elevate access and quality, the Ethiopian Ministry of Health devised a Public-Private Mix Implementation Guideline for health services in 2017. This initiative aimed to remediate irregularities in family planning service provision, addressing issues like insufficient contraceptives and provider comprehensiveness (168).

Ethiopia’s commitment to FP2020 and FP2030 initiatives, among other priorities, prioritized adolescents, regardless of marital status, introducing school-based family planning programs and a new cadre of health extension workers trained to serve youth (169, 170). The outcomes encompassed the Adolescent and Youth Health Strategy (2016–2020), enhancing adolescent information access and education while elevating the quality of youth-friendly health services (171), Actions for Acceleration (2018–2019) further aimed at curbing adolescent pregnancy, enhancing family planning among youth, with a particular focus on long-acting methods (172), and the A360 program focused on contraception among girls and young married couples (169, 173). Through a transdisciplinary approach, A360 merges public health, human-centered design, adolescent developmental science, socio-cultural anthropology, youth engagement, and social marketing, employing these diverse lenses to yield country-specific adolescent and youth sexual and reproductive health solutions (169, 174). In 2021, Ethiopia’s FP2030 commitment centers on augmenting family planning financing, securing contraceptive commodities, and bolstering adolescent access while ensuring quality information and service availability. Proposed strategies include fortifying supply planning, logistics management information systems, capacity building, and establishing youth-friendly health services. The strategies for enhancing adolescent services encompass improving provider knowledge, conducting advocacy campaigns against teenage pregnancy prevention, strengthening multi-sectoral coordination, and harnessing innovation and digital technology to enhance contraceptive access (175).

Despite concerted efforts to enhance access to sexual and reproductive health services for adolescents, negative attitude persists toward furnishing family planning services to unmarried adolescents (176). Enduring barriers include an unsupportive environment, negative attitudes of providers toward unmarried youth, supply constraints, poor implementation, inadequate multisectoral engagement, lack of parental and social support, gender inequality, and adolescents’ incomplete awareness and attitudes about sexual and reproductive health (136, 177). Additionally, within the HEP, the intersection of family planning and adolescents is maternal and child health, only when a married adolescent becomes pregnant (169).

While most contraceptives provided in the country came from donors and non-governmental organizations in earlier years, the trajectory changed in 2006 with a significant upswing in public health expenditure, soaring over 15-fold. By 2020, health spending peaked at US$ 3.63 billion, with reproductive health constituting 12.5% of total health expenditure, of which family planning accounted for 29% (approximately US$ 130 million) (178, 179).

### 3.5 Rwanda

Rwanda had a very low level of mDFPS up to 2000. From that point on, there was constant progress with mDFPS increasing from 18% in 2000 to 61% in 2010 and 73% in 2019. Over time, the disparity between the poorest and wealthiest groups gradually diminished. Starting in 2014, the coverage among the poorest surpassed that of the wealthiest, with the latter remaining stagnant at around 60%. The contraceptive method mix presented a very positive change too. The share of long-acting contraceptives increased from 6 to 51% in 2019, while permanent contraception reduced from 18 to 4%. The results confirm the assertion of the government of Rwanda as a pioneer in health reforms, considering family planning a top priority. Improving the quality of the family planning services was the major effort of the identified policies (Figure 5). A timeline encompassing key policies and programs enacted in Rwanda is presented in Table 5. A complete list of the identified policies is presented in Supplementary material.

**Table 5:** Timeline of the main policies for family planning in Rwanda.

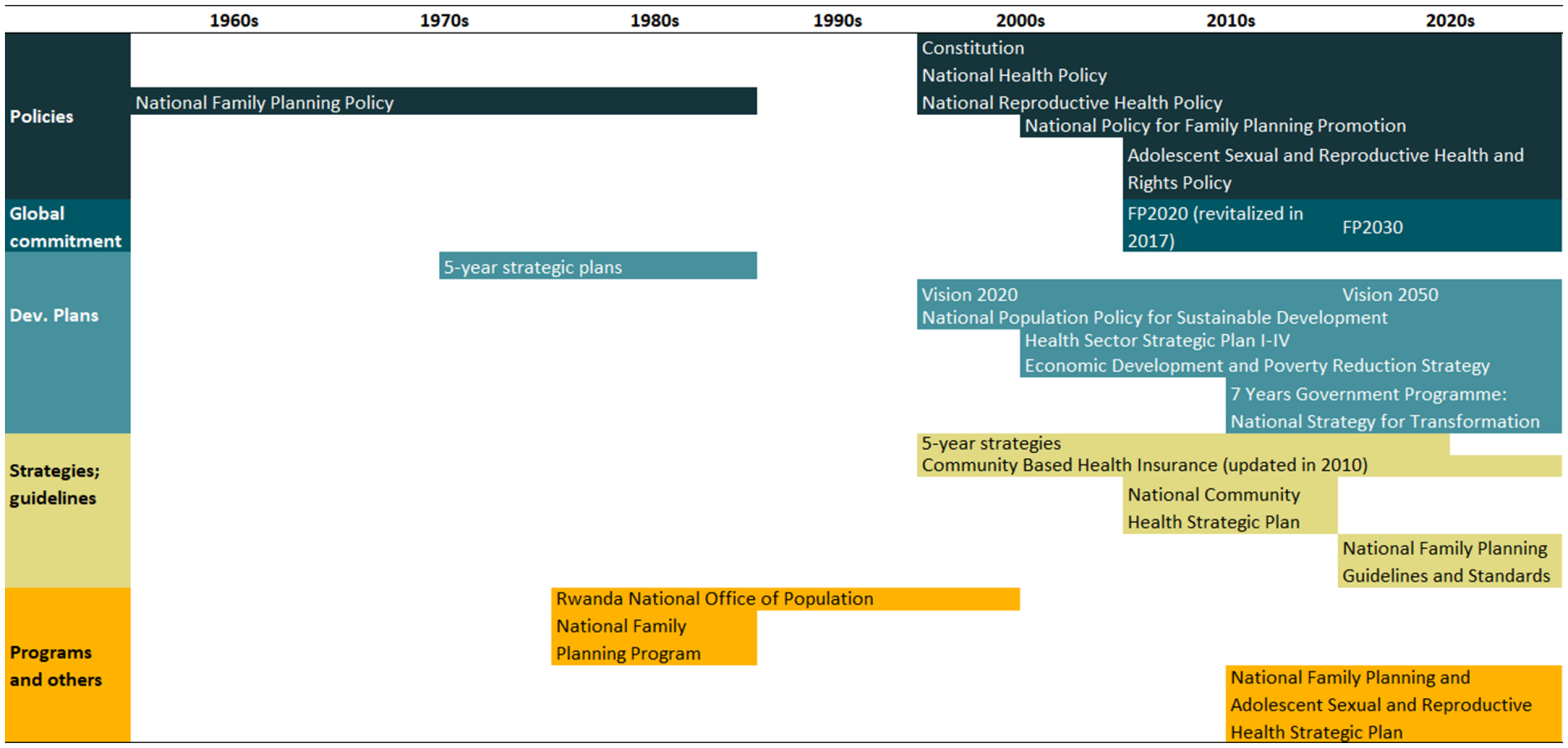

Aiming to reduce its fertility rate, the government of Rwanda implemented the first national family planning program in 1962 (180–182). Although the program included the provision of modern contraceptives, it primarily emphasized natural family planning approaches in line with cultural norms (181, 182). More concerted efforts took shape only in 1977 when family planning goals were embedded within the government’s inaugural 5-year strategic plan (1977–1981) (180, 181, 183).

With the mission of developing and implementing a national family planning policy, and promoting training of providers, the Rwanda National Office of Population (ONAPO) was created in 1981. Early projects of ONAPO were training programs organized for health agents to provide family planning services and education, with a focus on limiting childbirth. Concurrently, family planning services were integrated across all health facilities (8, 180, 181, 184–188). An instrumental project was the Information, Education, and Communication (IEC) program, which harnessed mass communication avenues such as radio, pamphlets, theater, cinema, meetings, and seminars to disseminate information on national development and demographic trends (188). Furthermore, the program trained couples to be family planning advocates, cascading the knowledge to other couples (188, 189).

In 1981, a new national family planning program was implemented, marked by the establishment of secondary family planning posts, provider training, improved supervision, consistent contraceptive supply, and method diversification (190).

In a context of high rates of illiteracy, and political and religious opposition, the use of modern contraceptives was still extremely low. However, the IEC program and the national family planning program spurred change. By the late 1980s, most of health facilities were offering family planning services, pharmacies were allowed to sell condoms, over 70% of the population knew about family planning, and new contraceptive users surged (180, 182, 188–192). ONAPO was responsible for the organization of several events to discuss obstacles to family planning use and the development of new strategies (184). However, although ONAPO has reached most of the population, it encountered public skepticism. By 1991, only 11% of married women had accepted contraceptive methods (187, 193). In 2003, ONAPO shuttered, passing population concerns to the Ministry of Health (181, 187, 194).

Other two 5-year strategic plans were developed in the 1980s. The 1982–1986 plan aimed to delay first birth and limit childbirth after age 40. To achieve this, new family planning services were made available and a campaign to educate the population regarding demographic issues was conducted (183). The subsequent 1987–1991 plan aimed at fertility reduction through family planning, providing training to family planning providers on interpersonal communication, coupled with the dissemination of demographic awareness and family planning benefits (183).

Ministerial Instruction No. 779 in 1988 advocated maternal-child health programs encompassing family planning. It advocated full integration of family planning services within health services, accompanied by guidelines on information provision, diverse contraceptive offerings, and continual health provider training (195, 196). An initiative from 1989 further integrated family planning into postpartum services, training providers to offer comprehensive information on various methods’ benefits, usage instructions, side effects, efficacy, and follow-up needs. Rwanda already boasted an array of modern methods such as pills, condoms, injectables, implants, IUDs, and sterilization. Providers were also trained to administer family planning services with sensitivity and respect (182).

The sequent 1990 National Population Policy aimed to intensify the efforts of IEC, and reduce fertility through the increase in contraceptive use (193). However, the 1994 genocide thwarted many planned actions, leaving the nation grappling with destroyed infrastructure, scarce medical supplies, and financial constraints. It led to a decrease in the prevalence of modern contraceptive use among married women from 13%, in 1992, to 4%, in 2000 (181). In the post-genocide years, population policy shifted focus toward reuniting dislocated families, resulting in a hiatus in family planning initiatives (182, 194).

In 2000, Rwanda’s government launched the Vision 2020 framework, presenting priorities for development. It became the guiding document for the development of future policies and programs. This framework recognized population growth as a vital challenge for economic advancement, thereby positioning family planning as a national priority (182, 197). Also in 2000, the Government introduced Community-Based Health Insurance (CBHI), covering over 95% of the population. Family planning services were among the health services more accessible through CBHI (198). The framework was later updated, with the launch of the Vision 2050 (199).

In 2003, the Constitution and the National Reproductive Health Policy solidified the groundwork for subsequent improvements. Both reinforced the principles of gender equality and affirmed citizens’ right to health. The National Reproductive Health Policy not only diversified family planning access through various health services but also bolstered provider competence through adherence to WHO guidelines, heightened contraceptive supply, and intensified efforts to advance women’s empowerment (200–202). The National Health Policy of 2004 further underlined family planning’s role as an important contributor to the health status of the family and pointed out the importance of integrating it with other services (201).

In 2005, the National Policy for Family Planning was launched along with its new 5-Year Strategy (2005–2010). Key elements of these policies were to mainstream family planning programs in all health services, to increase provision to a full range of methods, advocacy, community participation, public-private partnerships, sustainable financing of family planning, and evidence-based decision-making (200). An addition of this policy was the inclusion of the Standard Days Method, a cost-effective fertility awareness-based approach devoid of side effects, follow-up requirements, or faith-based group hesitations (203). The introduction of the method seems to have increased the contraceptive prevalence by appealing to couples new to family planning (204, 205). By 2010, contraceptive utilization had exceeded Ministry of Health’s targets, reflecting the huge success of the policy (202). Factors contributing to this achievement spanned supply and demand dynamics, encompassing performance-based financial schemes, amplified family planning services delivery by NGOs and private facilities, integration of family planning into common healthcare services, impactful mass media campaigns, and community health insurance (201). Despite the improvements promoted by this strategy, several points needed to be further explored, such as the availability of long-acting reversible methods and training for their insertion, initiatives targeting adolescents and men, augmented support for community health workers, and nationwide dissemination of family planning service guidelines and standards (201).

Simultaneously in 2005, the RAPID model was presented to parliamentarians (181, 206). The model projects the effect of rapid population growth across sectors and the benefits of family planning programs. It prompted a notable shift in government approach, transitioning from the negative implications of population growth to emphasizing the health and economic benefits of smaller families (181). Although the modern contraceptive rate had almost reached the 1990 level in 2005, important obstacles to delivering family planning services were identified. With more committed government support, several actions were taken to address the recognized barriers. These encompassed devising a national training curriculum for family planning providers, the provision of family planning services in remote locations by community health workers, organizing outreach campaigns, refining logistics systems, bolstering national and international budgets for contraceptive commodities to ensure diversity of contraceptive methods, and creating secondary health posts adjacent to faith-based health facilities not providing modern contraceptives. However, limited adolescent access to family planning services remained an unaddressed constraint in subsequent endeavors (181). These strategies significantly underpinned the remarkable increase in mDFPS and concurrent inequality reduction observed between 2005 and 2010. During this period, mDFPS increased from 16 to 61%, and the gap between the poorest and the wealthiest decreased from 20 to 10 percentage points (Figure 5).

To guide the health sector toward achieving Vision 2020 and the Millennium Development Goals, the Ministry of Health has been designing health sector strategic plans since 2005. The first Health Sector Strategic Plan (HSSP) 2005–2009 led to substantial improvements in health service accessibility and outcomes. For family planning, the subsequent HSSP (2009–2012) bolstered efforts and financial allocations. Measures were instituted to enhance reproductive health service quality and escalate family planning demand. These interventions were segmented into three tiers: family-based community services, population-oriented schedulable services, and individual-oriented clinical services (207, 208). This strength in the health system led to important improvements in health indicators, particularly for rural women living in poverty. Between 2006 and 2010, contraceptive prevalence surged by 350 and 150% in rural and urban regions, respectively (209).

The 2011 Adolescent Sexual and Reproductive Health and Rights (ASRH&R) Policy, along with its Strategic Plan, underscored the importance of furnishing adolescents with information, counseling, and access to FP methods. It also emphasized the creation of referral systems connecting health facilities and the community to enhance adolescent client follow-up (201). The third HSSP (2012–2018) concentrated on expanding ASRH&R, encompassing condom distribution across public and private sectors, bolstering access to long-acting and permanent methods, extending community-based family planning services, and fostering deeper collaboration with the private sector (201, 210). Additionally, a comprehensive sexuality education curriculum, introduced in schools in 2016, incorporated concepts related to sexual and reproductive health, including sexually transmitted infections, pregnancy, and contraceptive use (211). Despite these initiatives, contraceptive use remains low among unmarried adolescents (212, 213).

The Family Planning 5-Year Strategy (2012–2016) was built upon several other policies and strategies developed to promote the country’s socioeconomic development, such as the Vision 2020, the Economic Development and Poverty Reduction Strategy (2008–2012), and the National Population Policy for Sustainable Development 2003 (201). Its objective was to increase the use of modern contraceptives through a more supportive environment characterized by privacy and confidentiality, using evidence-based practices to stimulate demand for family planning and support effective supply (214). In addition, given the Vision 2020, the Ministry of Health has been working to decentralize the health system to bring services closer to the people (201). This decentralization drive extended to performance contracts forged between the central and local governments, aimed at enhancing social service delivery, including family planning. The community-based provision of family planning is a strong component of family planning programs, and it was reinforced in 2012–2016 strategy. It was promoted through the training of community health workers, logistic improvements, performance-based financing, and the integration of injectable contraceptives in their package (214). The package expanded in 2014 with the inclusion of Implanon in the method mix.

With a focus on community-based health services, the National Community Health Strategic Plan (2013–2018) was developed to guide the provision of sustainable quality and quantity health care services. To strengthen the community health services delivery, the policy aimed to improve the knowledge and skills of community health workers, increase the engagement of the community in community-based health programs, motivate them to provide family planning services, and bolster monitoring and evaluation activities (215). This strategy yielded positive outcomes, not just in servicing remote areas but also in ensuring contraceptive continuity and facilitating family planning discussions with husbands through community health workers (216).

The Health Sector Policy of 2015 was developed to ensure universal accessibility of equitable and affordable quality healthcare services for all Rwandans. It encompassed six priority areas, including family planning, intending to improve demand, access, and quality of essential health services. The policy also made efforts to strengthen the integration of maternal health with other health services (217). In the same year, Rwanda embraced the revised WHO recommendation on contraceptive use eligibility criteria, triggering an expansion of its Post-Partum Family Planning strategy and subsequently boosting family planning method uptake.

Recent policies comprise the National Strategy for Transformation, the fourth release of the HSSP, 2018–2024, the National Family Planning and Adolescent Sexual and Reproductive Health (FP/ASRH) Strategic Plan (2018–2024), its commitments with FP2020/FP2030, and the 2022 National Family Planning Guidelines and Standards. Enhancing the demographic dividend through universal access to quality healthcare stands out as one of the priority areas of the 7-year government program National Strategy for Transformation (2017–2024). To achieve this, the program aims to enhance healthcare facilities and expand initiatives aimed at promoting family planning and bolstering contraceptive utilization, particularly among adolescents (218). The HSSP IV is geared toward ensuring universal and equitable access to high-quality health services (219). It considers the lessons learned in the previous HSSPs and proposes interventions that are oriented to health services people-centered, integrated, and sustainable. Related to family planning, it seeks to incorporate family planning into antenatal and postnatal care and broaden social marketing of contraceptives, especially condoms, emergency pills, and long-acting reversible methods (219). The FP/ASRH Strategic Plan (2018–2024) is organized in a six components framework, considering the demand for FP/ASRH&R, supply of quality services, youth friendliness services, innovation, governance, data use and accountability, and policy environment (212). Rwanda’s FP2020 commitment underscored a focus on community health workers, long-acting and permanent methods, enhanced integration in health centers and hospitals, improved awareness, evidence-based practices, implementation of post-partum family planning services in health facilities, and improvements in family planning provision to adolescents (220). The FP2030 commitment highlights Rwanda’s dedication to enhancing service quality, diversifying contraceptive methods, employing new evidence-based strategies, augmenting the national budget for family planning, and employing performance-based financing (221). With a focus on quality of services, the recently disseminated National Family Planning Guidelines and Standards provides clear guidance for family planning programmers, public and private health facilities, and providers.

## 4 Discussion

This literature review encompasses the analysis of 196 policies enacted over more than 70 years across the five selected countries. We identified that in all countries selected, several laws, policies, and programs were implemented in a continuous and intertwined way, especially from the 1980s. Some are related directly to family planning, but others are more general and are aimed at primary health care or the health system as a whole. Additionally, we identified huge variations in the implementation strength of different programs. As all interventions were being implemented, simultaneous political and economic changes were unfolding.

Initiating their commitment to family planning in the 1960s, the five countries shared common efforts. The predominant focus of the policies across all countries was exclusively centered on women, with many policies focusing on the basic supply of contraceptives. Additionally, the five countries were committed to improving quality of care in family planning. This commitment encompassed the introduction of new contraceptives, the promotion of provider training, remodeling the model of care to a human-rights based approach, and the dissemination of family planning knowledge. Furthermore, the five countries implemented inclusive policies targeting the poor, rural, illiterate, and members of ethnic minorities. Ethiopia stood out with the most extensive array of policies. During the 2000s, civil society played an enormously important role in Ethiopia, actively crafting cohesive advocacy strategies to advance reproductive health within the country. The remarkable improvements were also attributed to the expansion and enhancement of primary healthcare units, as well as the implementation of the Health Extension Program. Efforts that bolstered healthcare accessibility and quality. Persistent challenges in Ethiopia are the negative attitude toward the provision of contraceptives to unmarried adolescents and the absence of family planning services for nulliparous adolescents within the Health Extension Program. Impressive efforts and progress were equally evident in Rwanda, attributable to a series of nationally instated policies by both the government and international partners. Rwanda’s strong political commitment drove a robust community-based program, the effective integration of family planning and maternal health services, and constant efforts to enhance family planning service quality. These efforts were further fortified by the establishment of a robust community health insurance framework. Collectively, these endeavors facilitated the expansion of family planning coverage and the reduction of related disparities. The introduction of new contraceptive methods and the availability of long-acting methods at lower-level health facilities, also provided by well-trained nurses and midwives, allowed the extraordinary change in the method mix we observe in the latest data.

Meanwhile, the Latin American countries, Brazil and Ecuador, achieved successful family planning outcomes through their public health systems, coupled with a consistent emphasis on gender equality and women’s health and rights. In Brazil, while arguments related to national development found limited traction, programs leveraged women’s health concerns, the family income-child ratio among the impoverished, and control over induced abortion (222). The country’s public health system allied with a constant focus on sexual, reproductive, and women’s health through a multitude of implemented policies and programs, has achieved huge success in family planning. Notwithstanding, there is still progress to be made, especially in achieving a more balanced distribution of contraceptive methods. Currently, long-acting reversible methods have limited participation, with cultural rejection, inadequate medical training, and constraints on healthcare personnel from insertion and counseling contributing to this situation (51). Ecuador’s progress in family planning during the examined period, particularly between 1994 and 2018, places it as a successful case in family planning. Family planning coverage increased and inequalities reduced dramatically. This positive trajectory coincided with policies acknowledging health as a fundamental human right, and prioritizing reproductive health through lenses of autonomy, gender equality, and societal engagement; in addition to strategies that guaranteed financial resources, social participation, and intersectoral collaboration.

In a context of a more conservative society and relatively limited gender equity (223), the progress observed in Egypt is important and laudable. The country achieved high levels of demand for family planning satisfied, with low levels of inequality in terms of several equity dimensions. This accomplishment can be attributed to the strong commitment of the government and the national and international organizations, and to their effort to be within the framework of the Egyptian culture. Nonetheless, Egypt charted its path, expanding family planning coverage through mass media campaigns and prioritizing fertility regulation. Except from the other countries, Egypt lacked policies specifically targeting adolescents or unmarried women. Recognizing the potential consequences of limited access to family planning services for these women is essential enhancing future interventions.

Attributing causality to interventions, even within clinical trials, poses a considerable challenge. Further complexities arise when examining the impacts of real-world policies. Although a causal relationship between the described policies and the family planning outcomes cannot be established, lessons appear from the similarities and differences identified in the five countries analyzed. An important common aspect is the integration of family planning within primary health care services so that access to family planning services is close to the users. A distinct characteristic that seems quite important is training community health workers in family planning, even if it is only to offer information. Community health workers are the closest link the women have to health services. They are usually from the community, and they are easier to access than nurses or doctors. Experiences like those from Ethiopia show that community health workers can be trained to deliver contraceptives, including long-acting reversible ones. This integration gives capillarity to the provision of contraception and makes it easier for women to seek help in case of any problems or difficulties they may experience.

Family planning services that are universal and cater to the needs of specific and difficult-to-reach groups are another common and important characteristic. Except for Egypt, all countries have programs aimed at adolescents and unmarried women who may have more difficulty accessing family planning services due to stigma or legal restrictions. Services where women feel comfortable, safe, and free of judgment are essential to reach young or unmarried women, and even married women who sometimes face opposition from family members to use contraception.

Another key aspect of all successful countries is the availability of family planning knowledge presented in a simple, honest, and understandable way, allied with efforts to provide a wide enough range of contraceptives that will match the needs of each woman. Additionally, in most countries, the constitution recognizes health and family planning as human rights, which paves the way for policies that guarantee access to health services and contraception that are free or subsidized so that these services are accessible and affordable.

On the other hand, many policies tend to focus on specific objectives like reducing fertility rates or increasing the uptake of modern contraceptive methods. This narrow focus often falls short of embracing crucial elements of a rights-based approach, such as informed choice and autonomy. Concentrating solely on specific targets may pressure family planning providers to meet those goals at the expense of individual women’s preferences, potentially hindering their ability to make fully informed decisions about family planning (224). Additionally, this approach often assumes that all women aspire to have smaller families and achieve this through the use of a modern contraceptive method, which may not always align with women’s actual desires (224).

The quality of services emerged as a recurring theme in the identified policies. Among the four characteristics we considered as related to enhancing the quality of family planning services, two that stood out were the training of healthcare providers and the introduction of new contraceptive methods. In most cases, these aspects were associated with the promotion of long-acting reversible contraceptives. While this aligns with a substantial body of literature that highlights their high effectiveness and high couple-years of protection, it is important to emphasize that a rights-based approach necessitates giving precedence to women’s preferences (224). Particularly in resource-limited settings, which often grapple with fragile supply chains, women’s choices may be influenced by the training of healthcare providers, which may prioritize one form of contraception over others (224). Within a rights-based framework, it is fundamental that policies acknowledge the necessity of making multiple contraceptive options readily available and address issues related to stock-outs of all contraceptive methods.

This study has several limitations. First, since Afghanistan was excluded, we could not get inputs from Asia, where many countries implemented important policies and reached high levels of family planning coverage. Secondly, data from national health surveys is available for a limited number of time points and, therefore, monitoring of the earlier and more recent progresses is not possible. The initial national health surveys were carried out in the 1990s and more recent surveys are not yet publicly available. Third, policies were not always clearly described, especially for the interventions implemented before the 1980s. This is due to the lower development of the information system and because the policymakers were less experienced at that point. Additionally, the occurrence of wars and national disasters resulted in the loss of important documents. In several cases, it was difficult to access their objectives, strategies, and results. Furthermore, within our study scope, emphasis was directed exclusively toward policies executed at the national and regional tiers, with policies at the state and municipality levels being omitted. It is undeniable that these policies could have contributed to the achievements in family planning coverage, especially in enhancing it in more vulnerable regions.

## 5 Conclusion

This literature review underscores that the progress observed in the selected successful countries happened in a context of cumulative and collaborative efforts involving governments, national and international organizations, and civil society. To expand family planning coverage, it is necessary to implement broader policies, that consider the diverse needs of the population and the specific contextual factors at play. Furthermore, the sustainability of these policies, coupled with economic growth and the allocation of resources, will significantly influence the outcomes of proposed healthcare initiatives.

We hope that this review will help countries grappling with the challenge of enhancing family planning coverage, whether on a national scale or among harder-to-reach subgroups. We hope it prompts stakeholders in the family planning community to critically assess the potential enhancements to their existing policies and identify new approaches tailored to the circumstances of each context.

## Author contributions

FH: Conceptualization, Data curation, Formal analysis, Investigation, Methodology, Validation, Visualization, Writing – original draft, Writing – review & editing. LM: Investigation, Writing – review & editing. MS: Writing – review & editing. CV: Writing – review & editing. PR-Q: Writing – review & editing. MM: Writing – review & editing. JS: Writing – review & editing. SR: Writing – review & editing. AN: Writing – review & editing. SM: Writing – review & editing. HR: Writing – review & editing. AB: Conceptualization, Data curation, Funding acquisition, Methodology, Project administration, Supervision, Validation, Visualization, Writing – review & editing.

## Footnotes

^1^https://www.who.int/publications

^2^https://fp2030.org/FP2030-commitment-makers

^3^https://www.unfpa.org/publications

^4^https://www.un.org/en/academic-impact/page/publications
