# Supplementary material for: Policies for expanding family planning coverage: lessons from five successful countries
Source: Front Public Health. 2024 May 14;12:1339725. doi: 10.3389/fpubh.2024.1339725 (PMC11131167; doi:10.3389/fpubh.2024.1339725)
Supplement: Supplementary file 1 [file Table_1.docx]

Supplementary Material

Figure S1. Trends in the prevalence of modern contraceptive use by country. The shaded area represents the 95% confidence interval. The lines start and stop at the year for the first and latest survey in each country.

**Supplementary Table 1.** Search strategy

| **Search** | **Database** | **Documents** |
| --- | --- | --- |
|  | **PubMed** |  |
| 2022-07-08 | (((("brazil"[Title/Abstract] OR "ecuador"[Title/Abstract] OR "egypt"[Title/Abstract] OR "ethiopia"[Title/Abstract] OR "rwanda"[Title/Abstract])) AND ((("family planning"[Title/Abstract]) OR ("contraception"[Title/Abstract])) OR ("reproductive health"[Title/Abstract]))) AND (("policy"[Title/Abstract] OR "intervention"[Title/Abstract] OR "evaluation"[Title/Abstract] OR "program"[Title/Abstract] OR "law"[Title/Abstract] OR "campaign"[Title/Abstract] OR "strategy"[Title/Abstract] OR "legislation"[Title/Abstract]))) AND (("1950"[Date - Publication] : "3000"[Date - Publication])) | 1,119 |
| 2023-07-05 | (((("brazil"[Title/Abstract] OR "ecuador"[Title/Abstract] OR "egypt"[Title/Abstract] OR "ethiopia"[Title/Abstract] OR "rwanda"[Title/Abstract])) AND ((("family planning"[Title/Abstract]) OR ("contraception"[Title/Abstract])) OR ("reproductive health"[Title/Abstract]))) AND (("policy"[Title/Abstract] OR "intervention"[Title/Abstract] OR "evaluation"[Title/Abstract] OR "program"[Title/Abstract] OR "law"[Title/Abstract] OR "campaign"[Title/Abstract] OR "strategy"[Title/Abstract] OR "legislation"[Title/Abstract]))) AND (("2022/07/08"[Date - Publication] : "3000"[Date - Publication])) | 82 |
|  | **Web of Science** |  |
| 2022-07-08 | **(((TS=(family planning)) OR TS=(contraception)) OR TS=(reproductive health) AND ((((TS=(brazil)) OR TS=(ecuador)) OR TS=(egypt)) OR TS=(ethiopia)) OR TS=(rwanda) AND (((((((TS=(policy)) OR TS=(inervention)) OR TS=(evaluation)) OR TS=(program)) OR TS=(law)) OR TS=(campaign)) OR TS=(strategy)) OR TS=(legislation) AND PY=(1950-2030))** | 1,833 |
| 2023-07-05 | **(((TS=(family planning)) OR TS=(contraception)) OR TS=(reproductive health) AND ((((TS=(brazil)) OR TS=(ecuador)) OR TS=(egypt)) OR TS=(ethiopia)) OR TS=(rwanda) AND (((((((TS=(policy)) OR TS=(inervention)) OR TS=(evaluation)) OR TS=(program)) OR TS=(law)) OR TS=(campaign)) OR TS=(strategy)) OR TS=(legislation) AND PY=(1922-2030))** | 323 |
|  | **SCOPUS** |  |
| 2022-07-08 | ( TITLE-ABS-KEY ( "brazil" OR "ecuador" OR "egypt" OR "ethiopia" OR "rwanda" ) AND TITLE-ABS-KEY ( "policy" OR "strategy" OR "program" OR "intervention" OR "evaluation" OR "law" OR "legislation" OR "campaign" ) AND TITLE-ABS-KEY ( "family planning" OR "contraception" OR "reproductive health") ) | 2349 |
| 2023-07-05 | ( TITLE-ABS-KEY ( "brazil" OR "ecuador" OR "egypt" OR "ethiopia" OR "rwanda" ) AND TITLE-ABS-KEY ( "policy" OR "strategy" OR "program" OR "intervention" OR "evaluation" OR "law" OR "legislation" OR "campaign" ) AND TITLE-ABS-KEY ( "family planning" OR "contraception" OR "reproductive health") ) AND PUBYEAR > 2021 | 78 |

Records identified through database Search (n=5,515)

Records excluded (n=3,324)

Wrong population: 258

Wrong outcome: 2,922

Studies proposing new strategies: 87

Text not available: 60

Records after duplicates removal (n=3,555)

Records screened (n=3,555)

Final studies included (n=231)

**Supplementary Figure 1.** Literature review flowchart.

**Supplementary Table 2.** Policies identified.

| **Country** | **Year** | **Policy** |
| --- | --- | --- |
| **Brazil** | 1965 | Foundation of the Brazilian Society of Family Welfare (BEMFAM) |
|  | 1974 | Foundation of the Centre for Research and Integral Care for Women and Children (CPAIMC) |
|  | 1974 | Establishment of INAMPS |
|  | 1975 | Second 5-year development Plan (1975-1979) |
|  | 1975 | National Maternal and Child Health Program (PMI) |
|  | 1980s | *Prev-saúde* |
|  | 1981 | Creation of the Brazilian Association of Family Planning (ABEPF) |
|  | 1983 | Program for Integrated Women's Health Care (PAISM) |
|  | 1985 | National Council for Women's Right |
|  | 1986 | Act of the Ministry of Social Assistance of Brazil to offer family planning services |
|  | 1988 | National Constitution |
|  | 1988 | PRO-PATER vasectomy campaigns |
|  | 1989 | Program of Adolescent's Health (PROSAD) |
|  | 1990 | Child and Adolescent Statute (Law 8069/1190) |
|  | 1990 | Unified Health System (Law 8080/1990) |
|  | 1991 | National Program of Community Health Agents (PNACS) |
|  | 1993 | Creation of the Cross-Sectoral Commission on Women's Health (CISMU) |
|  | 1994 | Family Health Program (PSF)/ Family Health Strategy (ESF) |
|  | 1994 | DKT social marketing of condoms |
|  | 1996 | Law Nº 9263 - "Lei do Planejamento Familiar" |
|  | 2004 | National Policy for Integrated Attention to Women's Health (PNAISM) |
|  | 2004 | National Pact for the Reduction of Maternal and Neonatal Death |
|  | 2005 | Law Nº 11.185 (update in the Child and Adolescent Statute) |
|  | 2005 | Adolescent Health Program |
|  | 2006 | Theoretical and Reference Framework: Sexual Health and Reproductive Health of Adolescents and Young People |
|  | 2006 | Regulation of the Medical Council Nº 1.811 (regarding emergency contraception) |
|  | 2006 | Levonorgestrel-releasing intrauterine system (LNG-IUS) Program of the International Contraceptive Access (ICA) Foundation |
|  | 2007 | Plan of Action (2004-2007) |
|  | 2007 | Health Legal Framework: an adolescent right |
|  | 2007 | National Family Planning Policy |
|  | 2008 | Program More Health: everyone's right |
|  | 2008 | Normative Resolution No. 167 of the ANS |
|  | 2009 | Adolescent Health Handbook |
|  | 2009 | Technical note Nº 13/2009 |
|  | 2010 | National guidelines for comprehensive health care for adolescents and young people in the promotion, protection and recovery of health |
|  | 2011 | National Program for Improvement of Access and Quality of Basic Care (PMAQ-AB) |
|  | 2011 | Stork Network |
|  | 2013 | National Plan of Policies for Women (PNPM) |
|  | 2017 | Ordinance Nº 3265 |
| **Ecuador** | 1965 | Foundation of the Association for the Well-being of the Ecuadorian Family (APROFE) |
|  | 1974 | Foundation of the Medical Center for Family Planning and Counseling (CEMOPLAF) |
|  | 1974 | National government officially adopts family planning |
|  | 1978 | Foundation of the Center for Studies in Population and Social Development (CEPAR) |
|  | 1994 | Free Maternity and Child Care Law |
|  | 1996 | National Quality Assurance Program |
|  | 2003 | The Children's and Adolescents’ Code |
|  | 2006 | Organic Law of Health (regulated in 2012) |
|  | 2006 | Update of the Free Maternity and Child Care Law (2006-2014) |
|  | 2007 | National Policy on Health and Sexual and Reproductive Rights (2007-2015) |
|  | 2007 | Action Plan for the Sexual and Reproductive Health and Rights Policy 2006-2008 |
|  | 2007 | National Plan for Pregnancy Prevention in Adolescents (2007-2010) |
|  | 2008 | Constitution |
|  | 2008 | Accelerated National Plan for the Reduction of Maternal and Neonatal Death (2008-2010) |
|  | 2009 | New Guidelines for Comprehensive Care of Adolescent Health |
|  | 2011 | Inter-Sectoral Strategy for Family Planning and Prevention of Adolescent Pregnancy (ENIPLA) (2011-2014) |
|  | 2013 | National Plan for Good Living (PNBV) (2013-2017) |
|  | 2015 | National Family Strengthening Plan Project (2015-2017) |
|  | 2017 | National Plan for Good Living (PNBV) (2017-2021) |
|  | 2017 | National Plan for Sexual and Reproductive Health (2017-2021) |
|  | 2018 | Intersectoral Policy for the Prevention of Pregnancy in Girls and Adolescents (2018-2025) |
| **Egypt** | 1961 | Creation of the Egyptian Family Planning Association (EFPA) |
|  | 1962 | Charter for National Action |
|  | 1964 | Creation of the Joint Committee for Family Planning |
|  | 1966 | Nasser-regime family planning program |
|  | 1966 | Creation of the Supreme Council for Family Planning |
|  | 1972 | Establishment of the UNFPA office |
|  | 1973 | UNFPA 1th Country Program for Egypt (1973-1977) |
|  | 1977 | Population and Development Program (PDP) |
|  | 1977 | Coptic Church Educational Program |
|  | 1978 | Strategy of National Development |
|  | 1983 | UNFPA 2th Country Program for Egypt (1978-1982) |
|  | 1979 | Family of Future (FOF) |
|  | 1979 | Establishment of the Information, Education and Communication (IEC) |
|  | 1980 | Egypt’s Family Planning Program |
|  | 1983 | UNFPA 3th Country Program for Egypt (1983-1987) |
|  | 1985 | Establishment of the National Population Council |
|  | 1985 | Population Policy |
|  | 1988 | UNFPA 4th Country Program for Egypt (1988-1992) |
|  | 1991 | Training program for family planning nurses |
|  | 1992 | Ministry of Health campaign to improve client satisfaction with family planning clinic services |
|  | 1993 | UNFPA 5th Country Program for Egypt (1993-1997) |
|  | 1994 | 20-year Program of action on reproductive health and rights |
|  | 1995 | Gold start program |
|  | 1997 | South-South cooperation |
|  | 1997 | Health Sector Reform Program |
|  | 1998 | UNFPA 6th Country Program for Egypt (1998-2002) |
|  | 2003 | UNFPA 7th Country Program for Egypt (2003-2007) |
|  | 2003 | “Your Health, Your Wealth” |
|  | 2008 | UNFPA 8th Country Program for Egypt (2008-2012) |
|  | 2013 | UNFPA 9th Country Program for Egypt (2013-2017) |
|  | 2016 | Sustainable Development Strategy: Egypt’s Vision 2030 |
|  | 2017 | Commitment with FP2020 |
|  | 2018 | Updated FP guidelines, following WHO's standards |
|  | 2018 | UNFPA 10th Country Program for Egypt (2018-2022) |
|  | 2022 | New initiative to support smaller families |
|  | 2023 | 11th UNFPA Country Program for Egypt (2023-207) |
| **Ethiopia** | 1966 | Foundation of the Family Guidance Association (FGAE), affiliated of the IPPF. |
|  | 1980 | Ministry of Health started to expand family planning services with the support of UNFPA. |
|  | 1981 | Creation of the National Office of Population (ONAPO) |
|  | 1984 | Establishment of the Cooperation for American Relief to Everywhere (CARE) |
|  | 1990 | Youth centers funded by IPPF |
|  | 1990 | Youth Counseling Services and Family Planning Education Project |
|  | 1990 | Initial DKT social marketing campaigns |
|  | 1992 | IPPF African Regional Workshop |
|  | 1993 | Health Policy of the transitional government |
|  | 1993 | National Population Policy |
|  | 1994 | Constitution |
|  | 1995 | Saturday Adolescent Family Planning/Counseling and Contraceptive Service (FGAE) |
|  | 1995 | Community-based distribution (CBD) / Community Based Reproductive Health Services (CBRHS) |
|  | 1995 | Essential Services for Health in Ethiopia (ESHE) Project (1995-2002) |
|  | 1996 | Guidance for Family Planning Services (updated in 2011) |
|  | 1997 | Information Education Communication/Advocacy Strategy |
|  | 1997 | Health Strategic Development Plan I |
|  | 2000 | Revised Family Code |
|  | 2002 | Health Strategic Development Plan II |
|  | 2003 | Second phase ESHE Project (2003-2008) |
|  | 2003 | Health Strategic Development Plan III |
|  | 2003 | Health Extension Plan |
|  | 2004 | National Youth Policy |
|  | 2005 | Plan for Accelerated and Sustained Development to End Poverty (PASDEP) |
|  | 2005 | Pathfinder International integrated youth-friendly services |
|  | 2006 | National Health Strategy (2006-15). |
|  | 2007 | National Adolescent and Youth Reproductive Health Strategy |
|  | 2008 | Population, Health and Environment Ethiopic Consortium (PHEEC) |
|  | 2008 | National Population Policy Plan of Action for 2008–2009 to 2015–2016 |
|  | 2009 | Government started to training health extension workers to the insertion of implants. |
|  | 2009 | Comprehensive Sexuality Education (CSE) policy |
|  | 2009 | Urban HEP |
|  | 2010 | Health Strategic Development Plan IV |
|  | 2011 | Global Health Initiative, implemented by USAID. |
|  | 2011 | Growth and Transformation Plan (GTP) |
|  | 2011 | Women’s Development Army |
|  | 2011 | Integrated Family Health Program (IFHP+) |
|  | 2012 | PPIUD included in the national training package for family planning |
|  | 2012 | Commitment with FP2020 |
|  | 2012 | School-based family planning program |
|  | 2013 | Link Up Project (2013-2016) |
|  | 2014 | Costed Implementation Plan for Family Planning 2015-2020 |
|  | 2015 | Postpartum Family Planning Action Plan |
|  | 2015 | Private Health Sector Project (PHSP) 2015-2020 |
|  | 2016 | Country Action Plan |
|  | 2016 | Adolescent and Youth Health Strategy (2016-2020) |
|  | 2016 | A360 |
|  | 2017 | Revitalized commitments with FP2020 |
|  | 2018 | Implementation of the Public–private partnerships in health framework |
|  | 2018 | Actions for Acceleration |
|  | 2021 | The Small, Happy, and Prosperous family in Ethiopia (SHaPE) |
|  | 2021 | Commitment with FP2030 |
| **Rwanda** | 1962 | Family Planning program |
|  | 1977 | 5-year plan (1977-1981) |
|  | 1981 | Creation of the National Office of Population (ONAPO) |
|  | 1981 | Information, Education, and Communication (IEC) program |
|  | 1981 | National Family Planning Program |
|  | 1982 | 5-year plan (1982-1986) |
|  | 1986 | ARBEF founded as local IPPF affiliated |
|  | 1987 | 5-year plan (1987-1991) |
|  | 1987 | Pharmacies were allowed to sell condoms |
|  | 1988 | Ministerial Instruction No. 779 |
|  | 1989 | Postpartum family planning program |
|  | 1990 | National Population Policy |
|  | 2000 | Rwanda Vision 2020 |
|  | 2000 | Community Based Health Insurance |
|  | 2002 | DELIVER project |
|  | 2002 | Standard Days Method pilot program, scaled-up in the following years |
|  | 2003 | National Reproductive Health Policy |
|  | 2004 | National Health Policy |
|  | 2005 | RAPID model presented to parliamentarians |
|  | 2005 | National Policy for Family Planning Promotion |
|  | 2005 | 5-year strategy (2005-2010) |
|  | 2005 | Health Sector Strategic Plan (2005-2009) |
|  | 2005 | Twubakane Program (12 districts) |
|  | 2005 | PSI Family Planning program |
|  | 2006 | Establishment of the pay for performance approach |
|  | 2006 | Capacity Project begins support for FP service delivery (11 districts) |
|  | 2006 | UNFPA begins support for FP (6 districts) |
|  | 2008 | Government Poverty Reduction Strategy (2008-2012) |
|  | 2009 | Second Health Sector Strategic Plan (2009-2012) |
|  | 2010 | Updated the Community Based Health Insurance |
|  | 2011 | Adolescent Sexual and Reproductive Health and Rights Policy |
|  | 2011 | Rio Political Declaration on Social Determinants of Health |
|  | 2012 | 5-year strategy (2012-2016) |
|  | 2012 | Third Health Sector Strategic Plan (2012-2018) |
|  | 2012 | Commitment with FP2020 |
|  | 2013 | National Community Health Strategic Plan (2013-2018) |
|  | 2013 | Economic Development and Poverty Reduction Strategy (2013-2018) |
|  | 2014 | Impano n’Impamba (“A gift for today that will last a long time”) program |
|  | 2015 | Health Sector Policy |
|  | 2015 | Constitution was revised |
|  | 2016 | Introduction of comprehensive sexuality education (CSE) in schools |
|  | 2017 | Revitalized commitment with FP2020 |
|  | 2017 | 7 Years Government Programme: National Strategy for Transformation (NST1) |
|  | 2018 | Fourth Health Sector Strategic Plan (2018-2024) |
|  | 2018 | National Family Planning and Adolescent Sexual and Reproductive Health (FP/ASRH) Strategic Plan (2018–2024) |
|  | 2020 | Rwanda Vision 2050 |
|  | 2021 | Commitment with FP2030 |
|  | 2022 | National Family Planning Guidelines and Standards |
